# A comb-brushing-type green soybean pod harvesting equipment: Design and experiment

**DOI:** 10.1371/journal.pone.0293567

**Published:** 2023-11-01

**Authors:** Ying Zhao, Jinyi Liu, Ranbing Yang, Ting Guo, Jian Zhang, Wen Li, Linji Li

**Affiliations:** 1 Mechanical and Electrical Engineering College, Hainan University, Haikou, China; 2 Hunan Tobacco Company, Chenzhou Branch, Chenzhou, China; NED University of Engineering and Technology, PAKISTAN

## Abstract

To solve the problem of low efficiency of manual harvesting of green soybeans and lack of adaptable harvesters, in this study, a brushing-type green soybean harvester was designed. The comb-brushing type green soybean pod harvesting equipment is composed of a front-mounted separation drum, a full-width material delivery mechanism, a negative pressure cleaning system, and a stalk-pod separation system. Based on the operation requirements of the front-mounted brushing-type detachment drum, the drum parameters, parameters of comb arrangement, and structural parameters of the comb, the force analysis in detachment was performed. By taking the pod detachment rate and damage rate as the response indexes, the rotational speed of the drum, the travel speed of the device, and teeth distance as influencing factors, a three-factor five-level orthogonal rotary combination test was carried out by the software Design-Expert. By establishing mathematical regression models for various influencing factors and evaluation indicators, conducting variance analysis and significance analysis on the response indicators of each factor, the optimal parameters were obtained at a rotational speed of teeth of 397.36 rpm/min, minimum axial teeth distance of 4.8 mm and travel speed of the device of 0.5 m/s. Field test results showed that, under the optimal parameter combination, the pod detachment rate was 94%, the damage rate was 3.04%, the harvesting efficiency was greater than 0.187 hm^2^/h, and impurity content was less than 7.8%, all of which met the design and usage requirements. The research results can provide a reference for the design of soybean harvesters.

## Introduction

**Fig 1:**
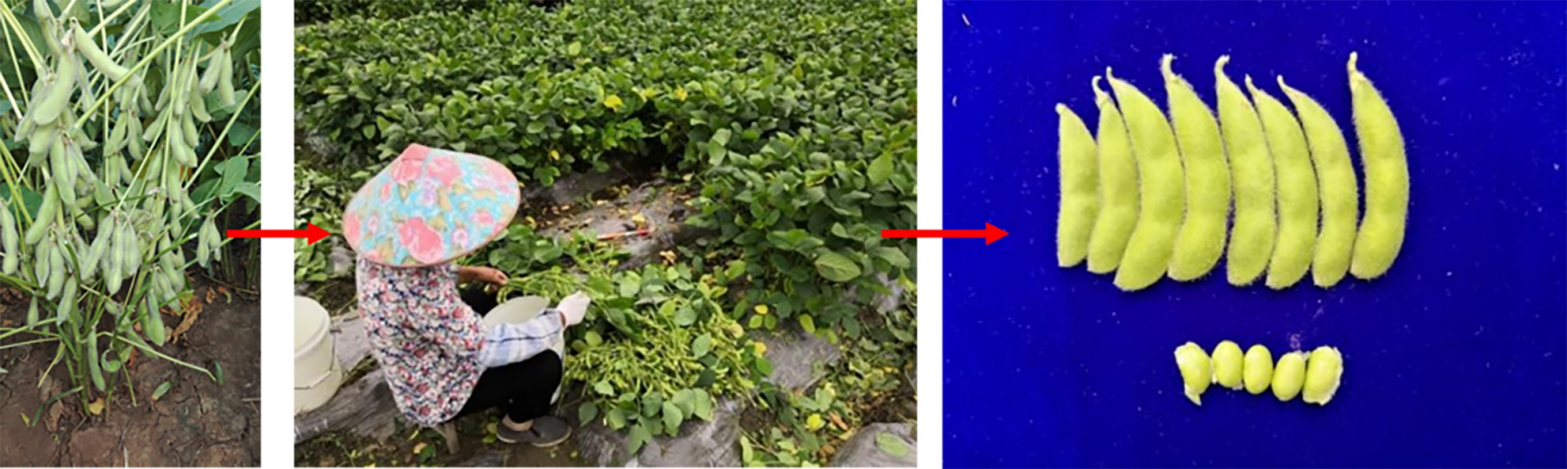
Manual harvesting process.

At present, the planting area of green soybeans in China is about 670,000 hm^2^, with an output of 10,500~12,000 kg/hm^2^ and a total output of 7,500,000,000 kg per year. Mechanization of green soybean planting, management, delivery, and post-harvest processing has been realized, but the harvesting takes up over 40% labor force of the whole production process and relies on manual work, the process is shown in Fig 1 [1, 2]. In China, green soybean pods serve not only as a directly consumable vegetable but also enable the preservation of seed freshness when harvested in this podded state. Since the harvest window for fresh green soybeans spans approximately ten days, the moisture content of these beans drops significantly after this period, thereby impacting their palatability. Given that manual harvesting allows for a maximum yield of 70–80 kg per day, a substantial amount of manual labor is required. Therefore, the design of a green soybean combine harvester is of great significance in improving the weak links in agricultural production.

During the harvest time of green soybeans, due to the obstacles of lush branches and leaves, high moisture content of beans (more than 70%), and plant lodging [3, 4], there are no mature green soybean harvesters in China. The experts in China studied the mechanical properties of green soybean plants, the harvesting mechanism, and the damage patterns of green soybeans. Zhao [5] studied the pod-stalk separation characteristics, designed a vertical roller mechanism in the green soybean separation testing device, and made an orthogonal regression test on its harvesting performance, then obtained the optimal parameter combination that affects the separation effect. Zhao et al. [6] studied the mechanical properties of green soybean pods and measured the elasticity modulus of green soybean seeds, pod shells, and pods and Poisson’s ratio of soybean seeds, and did finite element analysis of compression and verification test on B type and L type seeds and B type pods.

Developed countries started research on the threshing and cleaning of harvesters for these kinds of crops relatively earlier, and the harvesting and threshing methods mainly include the brushing and vertical roller method [7–12]. By the brushing type detachment method, the device harvests soybean pods through rotation and brushing of a front-mounted drum, on which the teeth are distributed regularly. The full-width material delivery mechanism has a rear-mounted grain-unloading mechanism, a negative pressure cleaning device, and a roller pod-stalk separation device, to realize continuous and mechanized harvesting of green soybeans. A typical harvester manufacturer of this method is GRIMME in Germany [13]. This kind of harvester using the brushing type method is applied in flat planting with a working width of 3.5 m and achieves a one-time harvesting rate of 89% and a damage rate of 18.0% [16]. It has a complicated structure with relatively advanced technologies, with functions of blade cleaning, steam cleaning, and hydraulic grain unloading, with easy operation skills. However, the width of the device is too large not suitable for the agronomic situation in China, and the damage rate is too high. The typical manufacturer of the harvester using the vertical roller harvesting method is JA (Japan Agricultural Co-operatives). The harvester made by JA harvests soybeans through rotation of double-rollers in a group of spiral rollers, and is suitable for single-ridge planting of green soybeans. When the harvester advances along the ridge furrow and after alignment, the green soybean plants are fed into two rollers. With the interaction effect between plants and harvesting rollers, the soybeans are harvested. After material delivery, blade cleaning, impurity removal, and pod collection, the mechanized harvesting of green soybean plants is realized [14]. The harvester using a vertical roller has the advantages of simple structure, one-stage sorting, detachment rate of 92%, and damage rate of 20.8%, however, it can only adapt to high ridge planting rather than flat planting and the damage rate is also too high. Moreover, without a grain unloading mechanism, the harvester should stop constantly, thus seriously affecting the working efficiency and farmers’ income [15, 16].

Currently, most existing soybean pod harvesters employ a combination of drum-type design with full hydraulic drive technology. However, these machines have not taken into account the mechanical characteristics of plants during pod harvesting, resulting in a relatively high rate of harvest losses [12, 16]. Additionally, previous research has not addressed the structural design for multi-row continuous harvesting. Moreover, the existing machines are of a large-scale structure, which is not suitable for the diverse planting characteristics in different regions of China.

Therefore, based on the comprehensive review of existing research, and building upon preliminary investigations, this study aims to establish a force analysis model for the brush-type separation of soybean pods and stalks. It will investigate the influence of operational parameters on pod separation efficiency and damage rate, optimize these parameters, and combine experimental results to refine the threshing drum design. The ultimate goal is to achieve the mechanized, multi-row, continuous harvesting of green soybeans, thereby enhancing the industrial competitiveness of pod-bearing legume harvesting machinery.

## Methodology

### The overall structure and working principles of soybean harvester

#### The overall structure of the equipment

**Fig 2:**
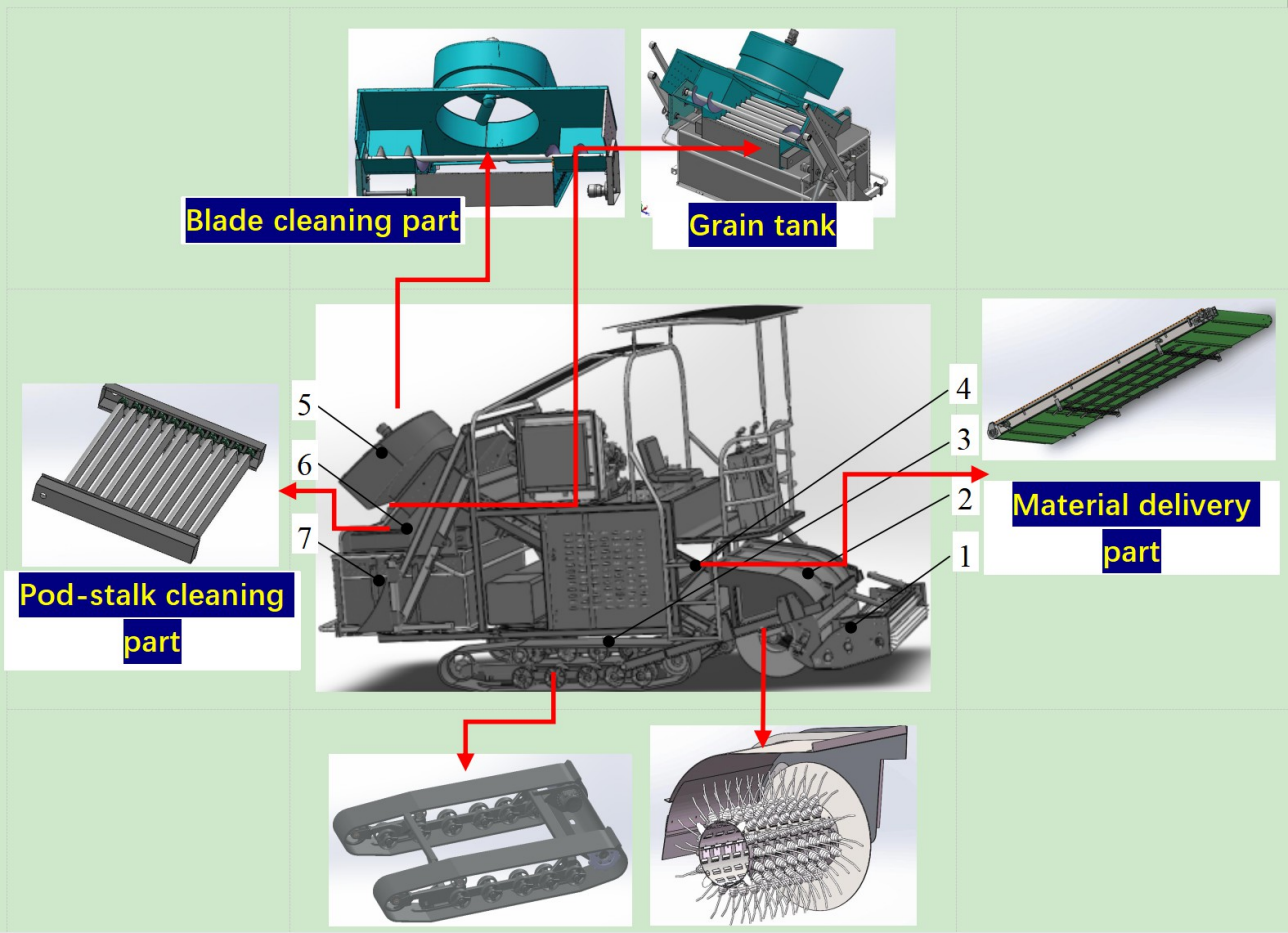
Structural diagram of the green soybean harvester. **Note:** 1- Reel part 2-A drum for separating pods from stalks 3- Chassis 4- Material delivery part 5- Blade cleaning part 6- Pod-stalk cleaning part 7- Grain tank.

The structure of the green soybean harvester was mainly composed of the straightening part, the pods and stalk separation mechanism, the material delivery part, the blade cleaning part, the pod-stalk cleaning part, the grain tank, the hydraulic system, and the chassis that holds all the functioning parts. Based on the arrangement of functional components on the chassis, the harvester features a front-suspension layout for the pod-stalk separation mechanism. The pneumatic components are integrated and designed at the rear. The material conveying component adopts a full-width mid-layout. The entire machine operates on a fully hydraulic drive system, with a closed-loop hydrostatic track-type drive for the chassis. The frame is constructed using integrated welding technology, and the cab is positioned on the upper supporting frame, providing convenience in operation and offering a wide field of vision. The schematic representation of the entire machine’s external structure is illustrated in Fig 2.

The operating procedures were as follows [17]:

1. Start the engine. The motor speeds of all working components can be adjusted through either the LCD touchscreen input or proportional valve pushrod control.
2. The hydraulic cylinders were individually controlled for their lifting and lowering through designated buttons.
3. During operation, set the grain-lifting components (Component 1) and the pod-stalk separation components (Component 2) to the working state. Ensure that the drum is no more than 10cm off the ground. Then, adjust the guide wheel to make contact with the ground. The walking operation of the harvester can be achieved by manipulating the hydraulic handle.
4. Activate the control motors for the other working components to set the harvester in motion.
5. When the grain box is full, use the grain box lifting cylinder and unloading plate to transfer the beans to the transport vehicle. Afterward, proceed to the next field for harvesting.

#### Working principle

**Fig 3:**
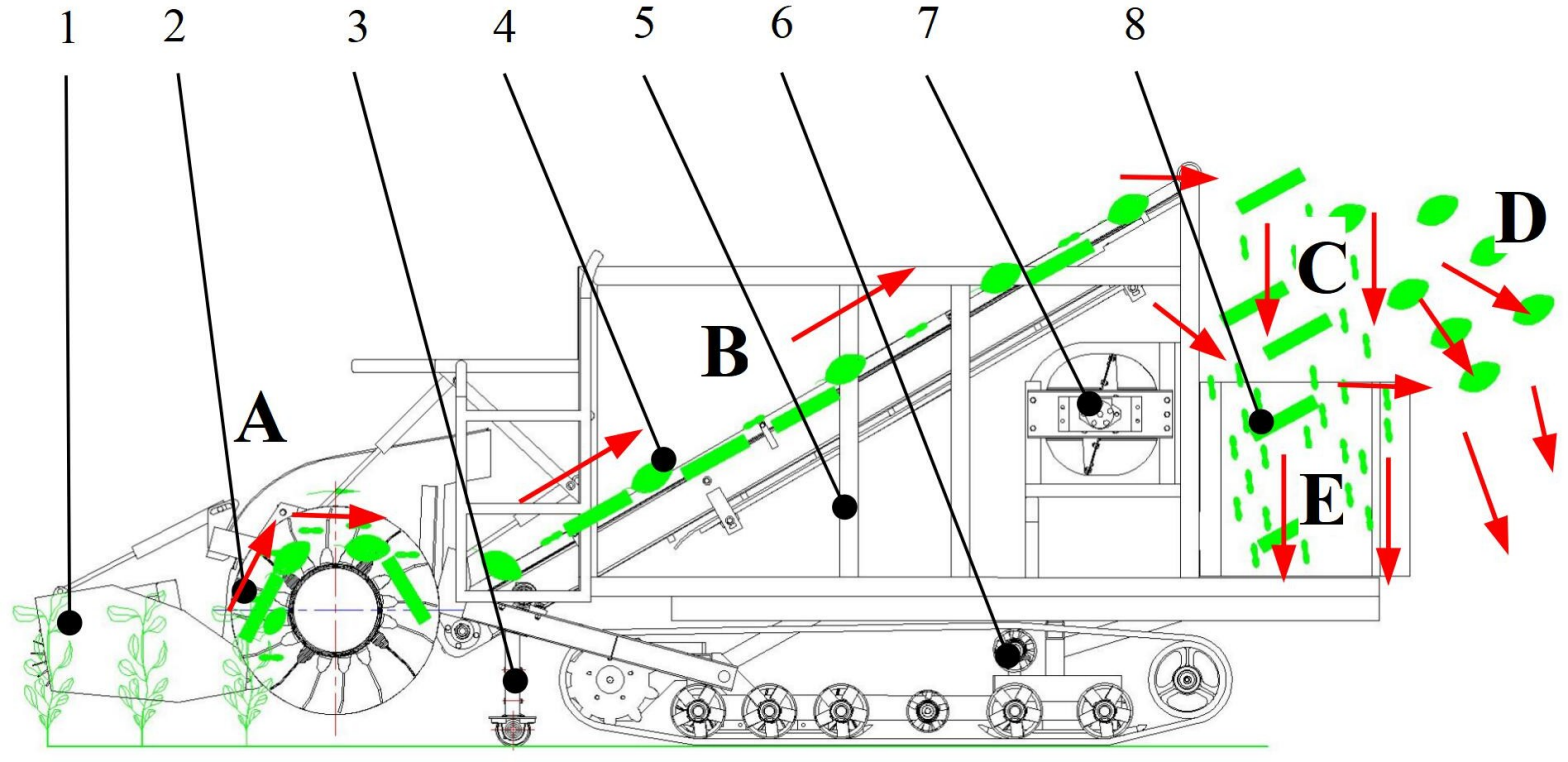
Principle of harvesting. 1. Green soybean plants; 2. Material granular particles inside the drum; 3. Contour wheel; 4. Chassis system; 5. Material granular particles in the delivery mechanism; 6. Frame assembly; 7. Lifting cylinder; 8. Pod unloading cylinder; 9. Material granular particles of the cleaning device; 10. Stalk and pod granular particles; 11.Leaf granular particles; 12.Pod granular particles; 13.Impurities. Note: The direction of the arrow was the direction of material flow.

The working principles for the green soybean harvester are shown in Fig 3. When the device advanced, the plants were divided into rows by the stalk divider, and then entered into the drum after they were pressed down by the reel brush, the flexible teeth on the drum were inserted into the plants. The high-speed rotating drum drives the flexible teeth to separate the bean pods from the stalks (position A); the materials after brushing were thrown backward to the trough plate delivery mechanism under the effect of inertia force (position B).

The materials were fed into the cleaning device through the conveyor belt (position C). The leaves are sucked into the fan enclosure by the high-speed axial flow fan (position D) and discharged in the field (position G). The stalks and pods are cleaned and separated at the cleaning part (position E), and the pods fall into the grain tank (position F) under the effect of gravity, and the stalks are discharged in the field through the cleaning part (position G).

### The key structural design of the drum mechanism for separating pods from stalks

#### Composition of the drum mechanism

**Fig 4:**
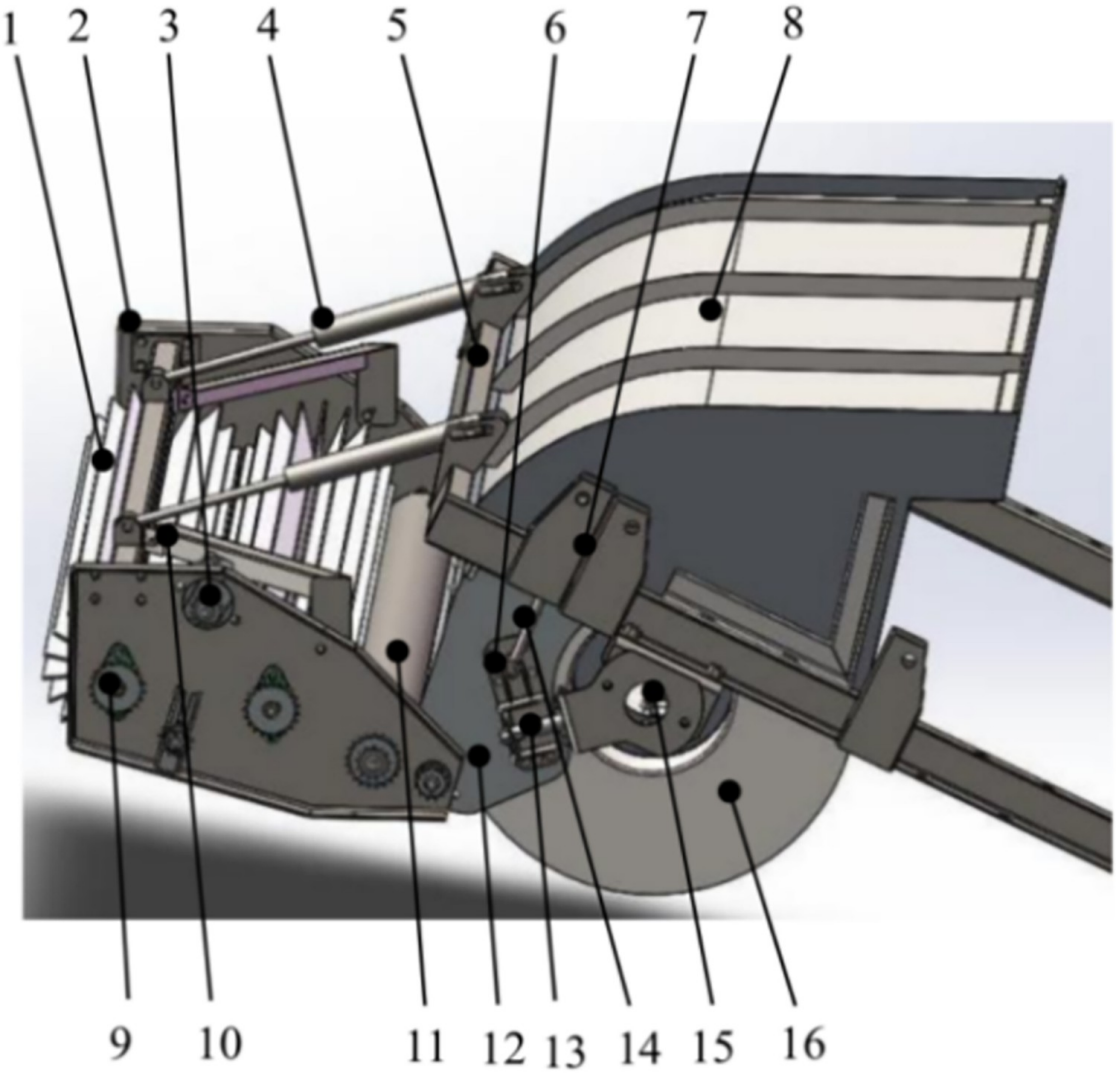
Schematic diagram of the drum mechanism for separating pods from stalks. 1. Reel; 2. Side enclosure; 3. Drive motor; 4. Lifting cylinder; 5. Bearing frame; 6. Support base; 7. Hanger; 8. Enclosure; 9. Drive sprocket assembly; 10. Reinforcing rib; 11. Roller; 12. Baffle; 13. Adjustment hydraulic cylinder I; 14. Adjustment hydraulic cylinder II; 15. Drum drive motor 16. Drum.

The schematic diagram of the drum [18] is shown in Fig 4.

#### Mechanical analysis in the harvesting process

The key component of the green soybean harvester was the drum mechanism, which directly impacts harvesting quality. This equipment encompasses three distinct processes: pod removal, pod rotation, and pod ejection. The primary factors influencing soybean harvest efficiency are as follows: in the pod ejection phase, the timing of comb teeth detachment from the plant, and the gap accounting for stalk brush misses. Entanglement may occur between the plant and the drum comb in the pod-carrying rotation phase. To mitigate these factors in the design, an analysis of parameters such as comb and stalk extraction speed, comb, and pod comb brush quantity, and comb and stalk extraction duration was imperative. Furthermore, it was crucial to determine parameters including drum mechanism height from the ground, shaft center height from the ground, rotational speed, tooth pitch, and comb length.

**Fig 5:**
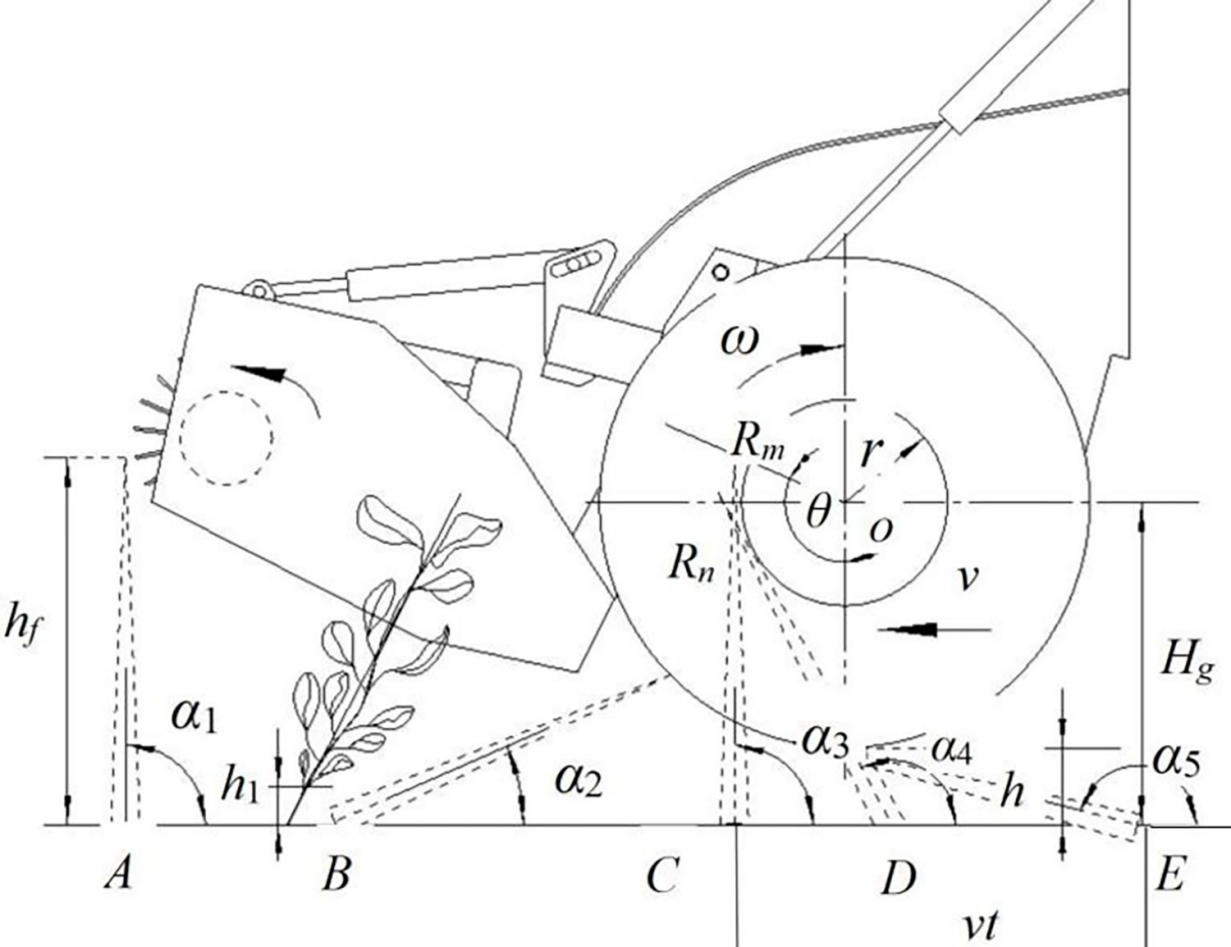
Schematic diagram of measuring points. **Note:** *h*_*f*_ is the height of soybean stalk, m; *α*_1_ is the angle between ground and plant before harvest, rad; *h*_1_ is the lowest distance from the ground to green soybean, cm; *α*_2_ is the angle between plants and ground in the feeding area, rad; *α*_3_~*α*_4_ is the angle between plants and ground in harvest area, rad; *α*_5_ is the angle between plants and ground in extraction state, rad; *h* is the distance of the drum to ground, cm; *v* is the travel speed of the device, m/s; *ω* is the rotational speed of drum, rpm/min; *r* is the inner diameter of the drum (mounting comb teeth), mm; *H*_*g*_ is the distance of drum axis from ground, cm; *θ* is the rotation angle of the drum, rad; *R*_*m*_ is the tangential point between the plant located at position C and the inner diameter of the roller; *R*_*n*_ is the tangential point between plant located at position *D* and the inner circle of drum; *vt* is the advance distance of *C* to *E*, m.

As shown in Fig 5, when the drum was working, the green soybean pods passed through positions *A*, *B*, *C*, *D*, and *E* in order to achieve the processes of harvesting, feeding, pod stripping, extraction, and stalks falling. In the extraction state, the drum was located at point *C*, and the stalk was tangent to the inner circle of the drum at point *R*_m_. Assume that at moment t, the angle between the central stalk and the horizontal plane was *α*_a_, point *O* was the axis of the drum, and the included angle between *O*-*R*_m_ and the vertical direction was *θ*. When the pods were brushed to point *D*, the top of the pod was tangent to the inner circle of the drum at point *R*_*n*_, and the extraction movement of the soybean stalk was completed. Where the stalk height was *h*_*f*_, the length of *C* to *R*_*m*_ was *l*_*cm*_, and the horizontal and left movement speed of the drum was *v*.

In the initial state of comb and teeth extraction, the length between the bottom of the soybean stalk and the point *R*_*m*_ was [17, 19]:

$$l_{cm}=\frac{H_{g}-r\cos\alpha_{a}}{\sin\alpha_{a}}$$ (1)

The length of straw wrapped on the drum was:

$$l^{\prime }=h_{f}-l_{cm}=h_{f}-\frac{H_{g}-r\cos\alpha_{a}}{\sin\alpha_{a}}$$ (2)

The winding angle of the stalk and the above winding length on the drum were:

$$\theta_{1}=\frac{l^{\prime }}{r}=\frac{h_{f}}{r}-\frac{H_{g}-r\cos\alpha_{a}}{r\sin\alpha_{a}}$$ (3)

At this time, the included angle *θ* between the tangent to point *m* and the vertical direction was:

$$\theta =\alpha_{a}+\theta_{1}=\alpha_{a}+\frac{h_{f}}{r}-\frac{H_{g}-r\cos\alpha_{a}}{r\sin\alpha_{a}}$$ (4)

It can be further calculated that the angular velocity of the comb-tooth pulling off the stalk tip was:

$$\frac{d\theta }{dt}=\frac{d\alpha_{a}}{dt}+\frac{H_{g}\cos\alpha_{a}-r}{r\sin^{2}\alpha_{a}}\frac{d\alpha_{a}}{dt}$$ (5)

If the angle between the stalk and the ground meets Eqs 6 and 7, the linear speed of the stalk tip extracted from the comb-tooth is shown in Eq 8:

$$\tan\alpha_{a}=\frac{H_{g}-r\cos\alpha_{a}}{vt-r(1-\sin\alpha_{a})}$$ (6)

$$\frac{d\alpha_{a}}{dt}=\frac{v\sin\alpha_{a}}{\cos\alpha_{a}(r-vt)-H_{g}\sin\alpha_{a}}$$ (7)

$$u=\frac{vr\sin\alpha_{a}}{\cos(r-vt)-H\sin\alpha_{a}}(r+\frac{H_{g}\cos\alpha_{a}-r}{\sin^{2}\alpha_{a}})$$ (8)

Eq 8 shows that the factors that affect the linear speed *u* were: the travel speed of the device *v*, the vertical distance between the drum and ground *H*_*g*_, and the radius of the drum *r*. In theory, a swifter detachment of the green soybean stalk resulted in a more pronounced impact on the pods. This led to an enhanced pod removal effect, reduced stalk entanglement, and minimized pod return loss [17].

Assuming that the time from the starting point *D* to the ending point *E* is Δ*t* and *l*_*cm*_ = *h*_*f*_ at the ending point *E*:

$$h_{f}=\frac{H_{g}-r\cos\alpha_{5}}{\sin\alpha_{5}}$$ (9)

Then, the time from angle *α*_3_ to *α*_5_ taken for the stalk to leave from the thumb drum was:

$$\Delta t=\frac{1}{v}[\frac{H_{g}-r\cos\alpha_{5}}{\tan\alpha_{5}}+r(1-\sin\alpha_{5})]$$ (10)

During comb-brushing, the pod-stalk detachment rate was in direct proportion to the number of brushing times *N*_0_. The larger the *N*_0_, the cleaner the pod will be stripped. The number *N*_0_ can be calculated from Eq 11.

$$N_{0}=\frac{m_{z}\omega }{60}\Delta t=\frac{m_{z}\omega }{60v}[\frac{H_{g}-r\cos\alpha_{5}}{\tan\alpha_{5}}+r(1-\sin\alpha_{5})]$$ (11)

Where *m*_*z*_ is the number of drum teeth, and *ω* is the rotating speed of the drum, rpm/min. In this formula, the travel speed increases, and the rotating speed of the drum also increases year-on-year.

Additionally, it is imperative to consider the geometric attributes of soybean stalks in the drum design. The rotational capacity of the pod should not only fulfill the comb-brushing prerequisites for entire stalks but also should guarantee that the pods located at the lowest parts of the plant are brushed. To enhance the comb-brushing effectiveness and diminish losses from stalk entanglement and pod fallback, the distance between the axis of the harvester and the ground should be increased as much as possible when designing the size parameters.

#### The determining parameters in drum mechanism design

**Fig 6:**
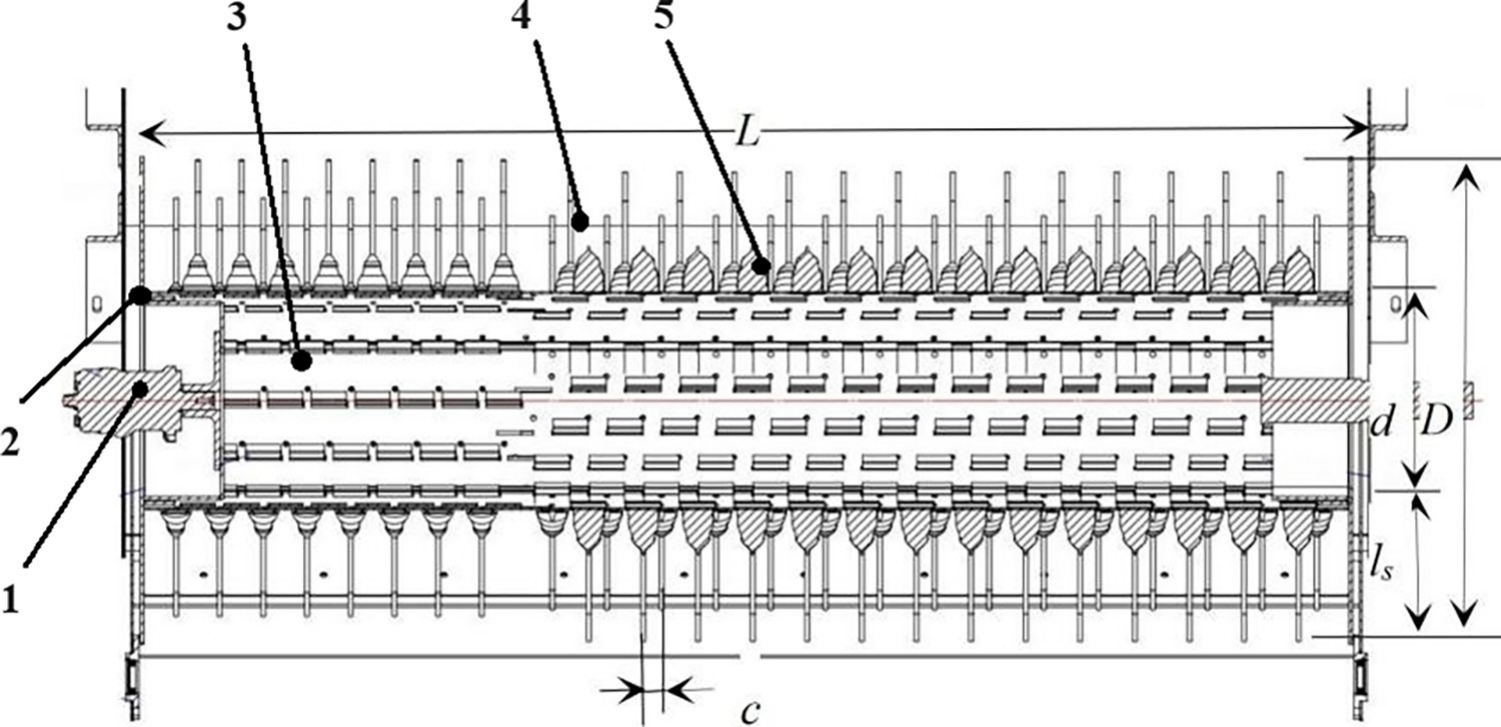
Schematic diagram of the drum. 1. Driveshaft; 2. Drum enclosure; 3. Drum; 4. Teeth; 5.Riveting slot. Note: *d* is the inner diameter of the drum (mm); *D* is the outer diameter of the drum (mm); *L* is the length of the drum (mm); *l*_*s*_ is the length of a single tooth; *c* is the minimum teeth distance (axial direction of the drum).

The structure of the drum mechanism is shown in Fig 6. It is mainly composed of a drive shaft, a drum enclosure, a drum, and teeth. The teeth were uniformly distributed on the drum in riveting slots. The drum takes off the green soybean pods by its teeth during rotation [20]. The drum was made of Q235 material [22]. The processing of the drum body involved laser cutting, followed by CNC bending and welding to achieve the final form. Subsequently, the comb teeth were uniformly installed on the surface of the drum through riveted slots. After installation, the drum must undergo dynamic balancing testing. Only when the test was passed can the drum mechanism be installed on the whole equipment.

The diameter and length of the drum were closely related to harvesting quality and harvesting width. The calculation method of the diameter of the drum was:

$$D=d+2l_{s}$$ (12)

Where, *D* was the outer diameter of the drum, mm; *d* was the inner diameter of the drum, mm.

According to literature [21], the diameter of the drum was generally 550~680 mm. Considering that the fresh green soybean plants have high moisture content with lush leaves and the material density in the drum was too high in the brushing process, therefore, due to the power consumption of the drum and installation space, the outer diameter of the drum was determined to be 670 mm, the inner diameter was 300 mm, and the teeth length was 185 mm.

The drum length corresponds precisely to the operational width of the harvester. Currently, fresh green soybeans are predominantly planted in three or four rows, with a ridge width of 1600 mm. For harvesting crop in 3 rows, the ridge width was 1600 mm with a row distance of 600 mm and a side distance of 200 mm; for harvesting crop in 4 rows, the ridge width was 1600 mm with a row distance of 450 mm and a side distance of 125 mm. Taking into account the current status of agronomic planting, *L* was determined to be 1600 mm.

The calculation of the rotating speed of the drum was:

$$\omega =6\times 10^{4}\frac{v_{g}}{\pi D}$$ (13)

Where, *v*_*g*_ was the linear velocity of the teeth on the drum, m/s.

Upon contact between the soybean pod and the teeth of the drum, the pod experienced an outward linear velocity, denoted as *v*_g_, due to the impact of the teeth. In a very short time interval Δ*t*, the pod’s velocity rapidly increases from 0 to *v*_g_. The kinetic energy of the teeth was transferred to the pod. Assuming the pod undergoes a substantial impact force *F*_*n*_., according to the impulse theorem, this impact force can be calculated.

$$F_{n}=\frac{m_{g}v_{g}}{\Delta t}$$ (14)

Where, *F*_n_ was the impact force on the pods, N; *m*_*g*_ was the weight of the pod, kg; Δ*t* was the contact time between teeth and pods, s.

**Fig 7:**
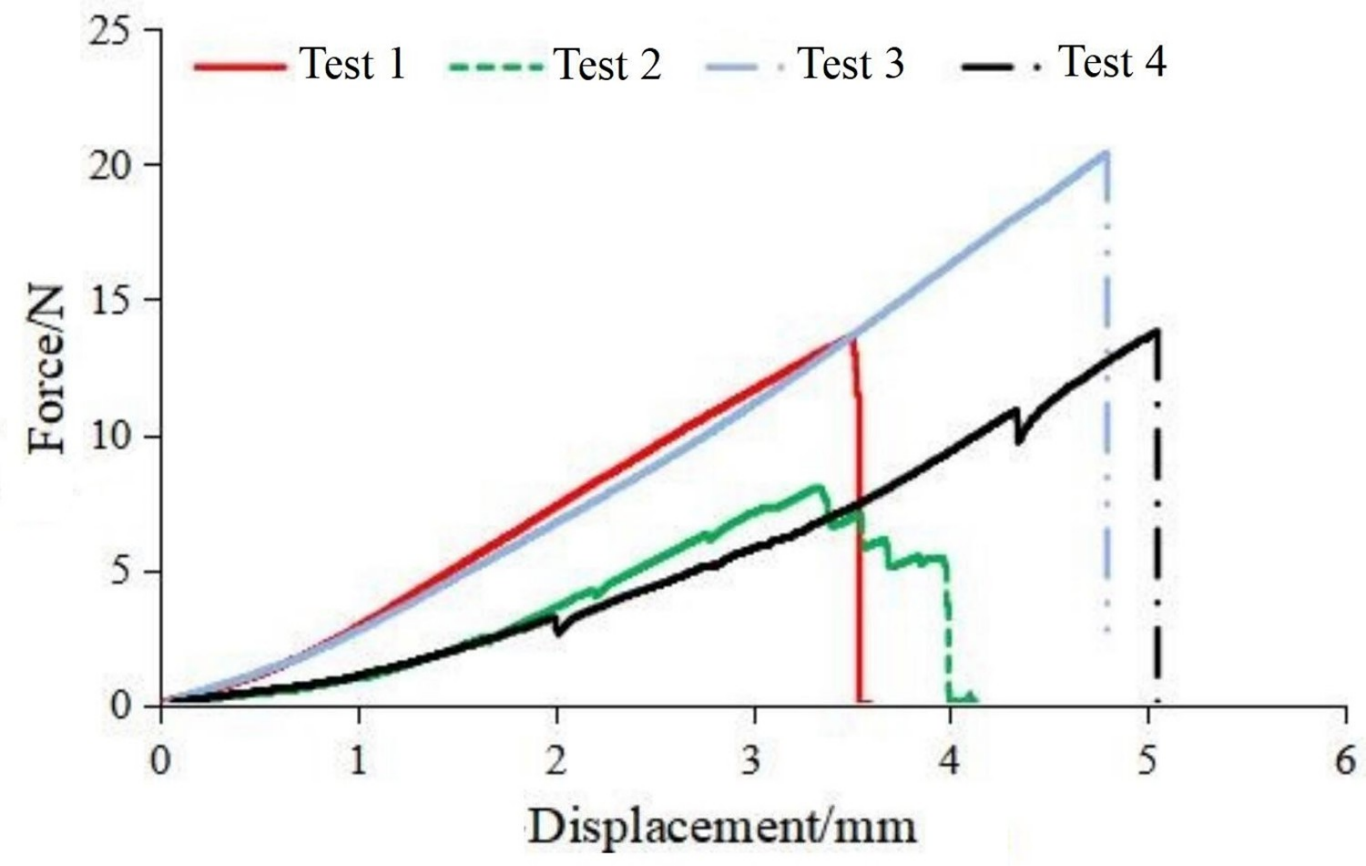
Pod stalk tension-displacement curve.

**Fig 8:**
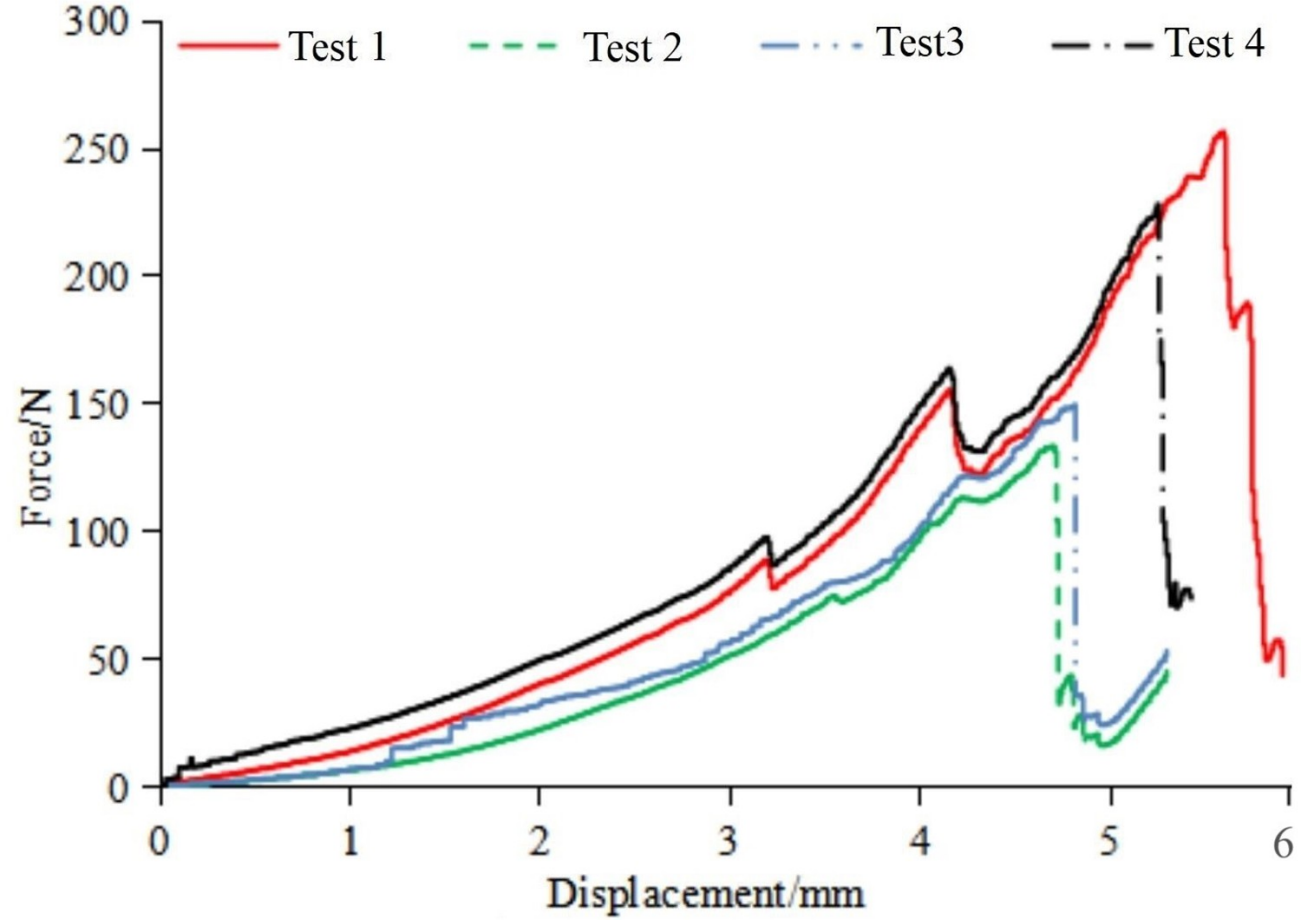
Pressure-displacement curve of the pods.

The mechanical properties of soybean pods are characterized by pod detachment strength, compression strength, elastic modulus, and elongation at break. This paper primarily focused on pod detachment strength and compression strength. Pod detachment strength determines the force required for soybean pods to detach from the stalk. Compression strength refers to the ability of soybean pods to withstand pressure and the force at which they will fracture under compression. The test material employed in this study was ‘Dou Tong No. 6,’ with an average stalk moisture content of 75.8%, grain moisture content of 80.5%, and pod shell moisture content of 72.7%. The soybeans of this variety were subjected to a tensile test for pod detachment from the stalk using a universal tensile testing machine. Additionally, a compressive test was performed on the pods using a universal compression testing machine. This yielded the force-displacement curves for pod-stalk separation and pod breakage, as shown in Figs 7 and 8, respectively. As observed in the tensile test of the pod stalk in Fig 7, its mechanical properties exhibit linear variation. When the applied force exceeded its critical force, the pod stalk was fractured, resulting in pod detachment occurring within the range of 5 to 30 N. In the compressive test of the soybean pod depicted in Fig 8, its mechanical behavior demonstrates non-linear variation. At a compression load of 150 N, the soybean pod initially incurred damage while maintaining elastic mechanical characteristics. However, when the applied load exceeded 250 N, the soybean pod experienced a complete rupture.

To ensure pods have no damage, the force *F*_*n*_ should be satisfied the scope.

$$F_{t}\leq F_{n}\leq F_{p}$$ (15)

Substitute Eqs (13) and (14) into Eq (15), and the following can be obtained after calculation:

$$\frac{6\times 10^{4}F_{t}\cos\xi \Delta t}{m_{g}\pi D}\leq n_{z}\leq \frac{6\times 10^{4}F_{P}\cos\xi \Delta t}{m_{g}\pi D}$$ (16)

Where, *F*_*t*_ is the force of separating pods and stalks, which was among 5~30 N, and took the maximum value of 30 N; *F*_*P*_ is the pod damage force, which was between 150~200 N, and took the minimum value of 150 N; *m*_*g*_ is the weight of a single pod, which was 2.78 g; *D* is the outer diameter of the drum, which was 670 mm; The angle *ξ* represents the angle formed during the harvesting process due to the mutual collision of pods, considering cos*ξ* as the collision loss coefficient of pods, usually taken as 0.5; Δ*t* was obtained through high-speed photography, whose value was 6.498×10^−4^ s, the rotational speed of the drum was calculated as 33.33 rpm/min^-1^≤*ω*≤499.97 rpm/min. Considering the special situations in field harvesting, the maximum rotational speed of the device should have a 20% design margin, therefore, continuously variable hydraulic transmission was employed to achieve a drum rotation speed range of 0 to 600 revolutions per minute (rpm).

#### Arrangement of comb teeth

**Fig 9:**
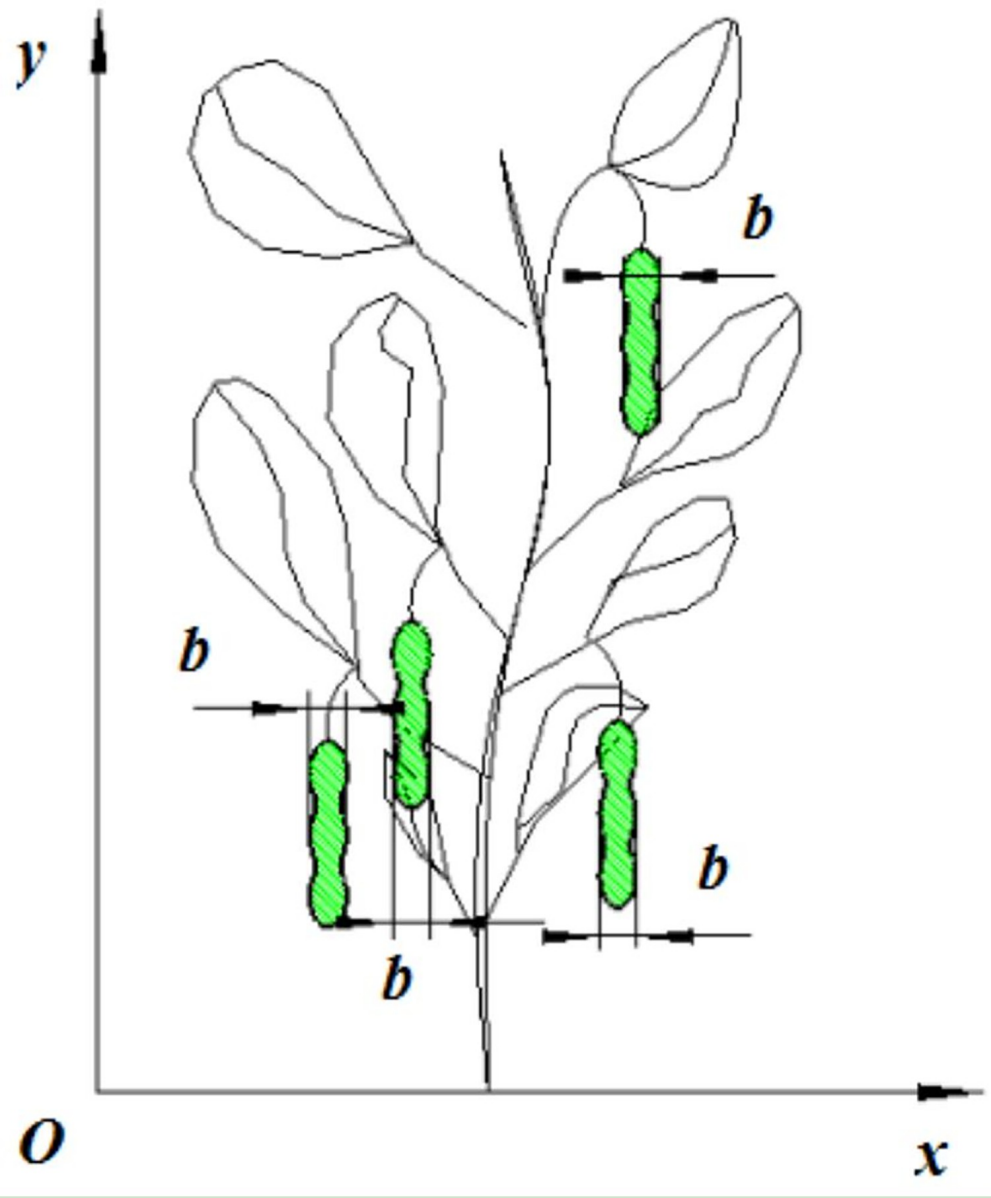
Teeth arrangement and plant combing and feeding. Note: *b* is the width of a green soybean pod, mm.

Within the drum’s designed structure, the pivotal components for effective harvesting were the comb teeth. Their arrangement played a crucial role in governing detachment and damage rates. It was imperative to ensure uniformity in the total impacts and their frequency from the moment of feeding pod particles to the actual pod removal process. As depicted in Fig 9, it provides a visual representation of the teeth arrangement, plant combing, and feeding process. Throughout the drum’s rotation, each pod should experience multiple impacts from the teeth. Specifically, within interval *b*, a pod must encounter at least one impact from the teeth per rotation of the drum.

**Fig 10:**
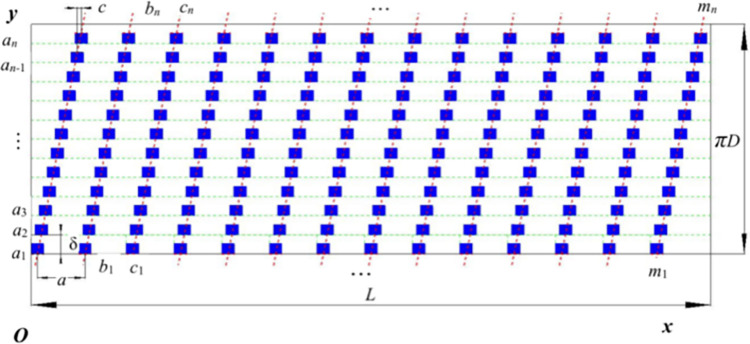
Expanded view of the drum. Note: *L* is the length of the drum; *πD* is the perimeter of the drum; *a*_*1*_, *a*_*2*_, *a*_*3*_, …, *a*_*n*_ are in the first column in the radial direction of the drum; *b*_*1*_, *b*_*2*_, *b*_*3*_, …, *b*_*n*_; *m*_*1*_, *m*_*2*_, *m*_*3*_, …, *m*_*n*_, which are in the second and the *m-th* columns, respectively; *a* is the axial distance of two neighboring teeth; *δ* is the radial distance of two neighboring teeth after unfolding the drum; *c* is the minimum distance of two neighboring teeth in the radial direction.

The expanded view of the drum is shown in Fig 10. The horizontal center line of the axle diameter was taken as the axial, and the travel direction of the harvester was taken as the radial, *c* was the minimum distance of two neighboring teeth in the radial direction, the distance between two neighboring teeth in the direction of axial was *b*. *L* was the length of the drum, *a* was the teeth space in the direction of the axial. From the shaft end direction of the drum, the distribution of teeth after rotating clockwise is: *a*_1_, *a*_2_, *a*_3_, …, *a*_*n*_, from the horizontal overview of the drum, the distribution of teeth is: *a*_1_, *b*_1_, *c*_1_, …, *m*_1_, the distribution of teeth on circle *b*_1_ is *b*_1_, *b*_2_, *b*_3_, …, *b*_*n*_, and the distribution of teeth on circle *c*_1_ is *c*_1_, *c*_2_, *c*_3_, …, *c*_*n*_, and so on. The distribution of teeth on circle *m*_1_ is *m*_1_, *m*_2_, *m*_3_, …, *m*_*n*_. *c* is the arc length of the drum after unfolding it in the direction of the radial, *πD* is the perimeter of the drum [22, 23].

According to Fig 10, the number of teeth in the radial direction can be calculated as follows:

$$n=\frac{\pi D}{\delta }$$ (17)

Where, *δ* was the radial distance of two neighboring teeth after unfolding the drum, generally its range was 18~250 mm (the arc length). Considering the installation size of teeth and the dynamic balance of the drum, *δ* was determined to be 105.2 mm and the number of teeth *n* in the radial direction was 20.

The arrangement of teeth should satisfy the requirement of Eq (18), that is, when the drum makes a revolution, in Fig 10, there was at least one impact from the teeth in interval *b* [24].

a=K⋅c⋅n (18)

Where *a* was the distance between two neighboring teeth in the axial direction; *K* was the number of impacts between teeth and pods, which was 1. *C* was the minimum distance of two neighboring teeth in the axial direction, which was pod width and the value was 5 mm; *n* was the number of teeth in the radial direction, and the value was 20; and the distance of teeth in the axial direction *a* was 100 mm.

L=a⋅m (19)

Where, *L* was the distance of the drum in the axial direction, which was also its width and the value was 1600 mm. The parameter *a* was the teeth distance in the axial direction, the value was 100 mm, and the arrangement of *m* in the radial direction was 16.

#### Determine the structure parameters of the teeth

**Fig 11:**
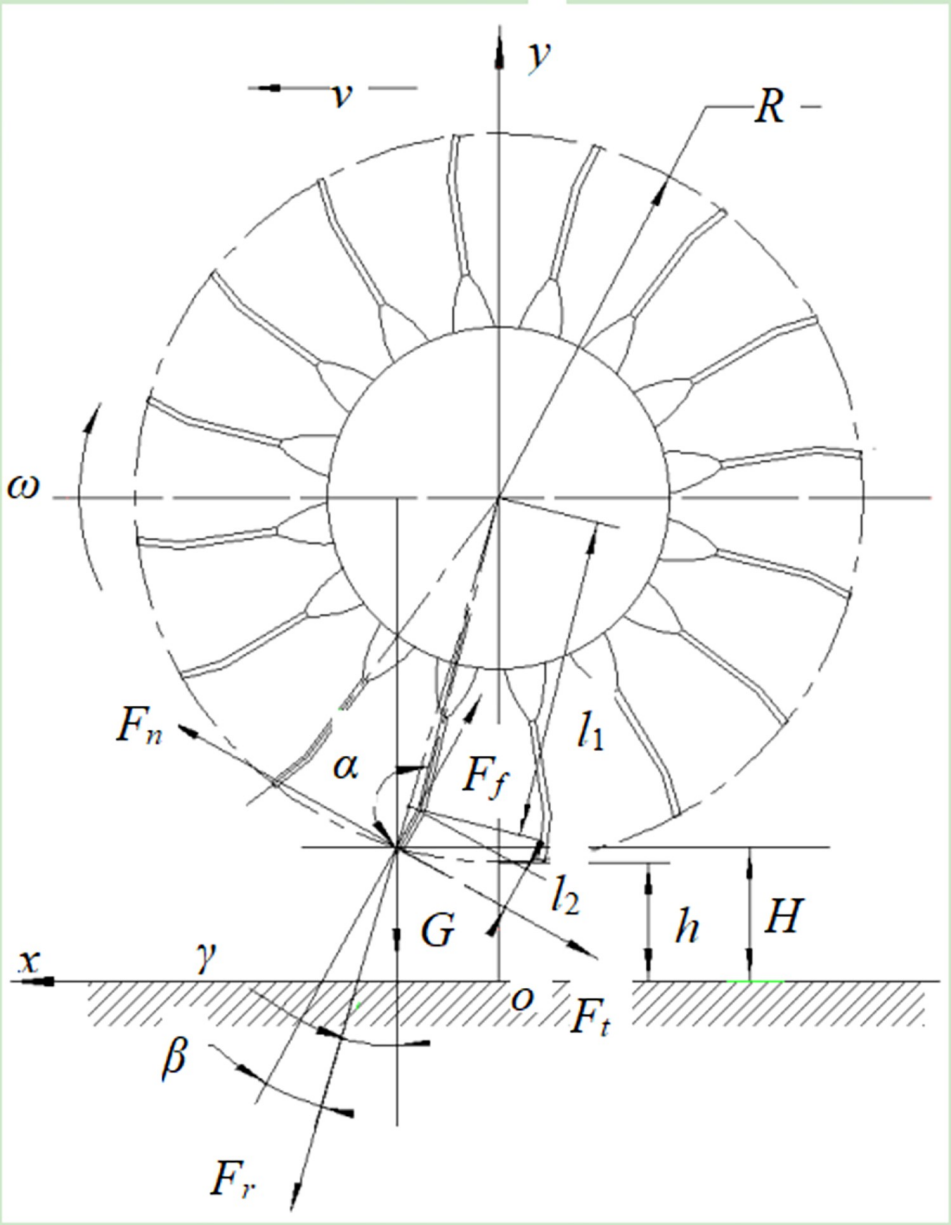
Kinematic analysis on the collision between materials and the teeth. Note: *R* is the outer diameter of the drum, mm; *v* is the travel speed of the device, m/s; *F*_*f*_ is the friction when throwing back the green soybeans, N; *h* is the minimum drum-ground distance, mm; *H* is the drum-ground distance when the teeth start to hull soybeans, mm; *G* is the gravity of pods, N; *β* is the angle between the bending section of the comb and the centrifugal force, (rad); *F*_*r*_ is the centrifugal force of the soybean pods, N, *γ* is the angle between the gravity of pods *G* and the centrifugal direction of *F*_*r*_, (rad); *F*_*n*_ is the collision force from teeth to pods, N; *l*_1_ is the distance between the bending section of the teeth and center of the drum, mm; *l*_*2*_ is the length of the bending section of the teeth, mm.

Bending the teeth of the drum by a specific angle facilitates the efficient ejection and rebound of detached soybean pods, thereby enhancing the detachment rate. By conducting a force analysis on the interaction between the comb teeth and the separation process of soybean pods and stalks, an optimal bending angle for the comb teeth can be calculated. Given that the size of the soybean pods was much smaller than the rotational radius of the drum, the pod can be approximated as a point. When the pod was lifted by the resilient teeth, its force distribution was illustrated in Fig 11.

To ensure that the soybean pods, after detachment, smoothly enter the conveying mechanism rather than falling to the ground inside the drum, the friction between the soybean pod material and the comb teeth must exceed the sum of the gravitational force and the centrifugal force acting on the soybean pod material at the end of the comb teeth. This can be expressed as the force condition for the soybean pod, as shown in Eq 20.

$$F_{f}\geq G\cos(\beta +\gamma )+F_{r}\cos\beta$$ (20)

Where,

$$F_{f}=\mu F_{n}$$ (21)

$$F_{r}=mR\omega^{2}$$ (22)

$$F_{n}=G\sin(\beta +\gamma )+F_{r}\sin\beta +F_{t}$$ (23)

Where *F*_*f*_ was the friction on green soybeans when throwing them backward, N; *G* was gravity of green soybeans, N; *β* was the angle between the bending section of the teeth and the centrifugal force, (rad); *F*_*r*_ was the centrifugal force applied to the green soybean plant when it was lifted, N; *γ* was the angle between the gravity of the pods and the centrifugal direction, (rad); *μ* was the friction coefficient between the pod plant and the teeth; *F*_*n*_ was the thrust exerted by the teeth when the fresh green soybean plant was picked up, N; *F*_*t*_ was the pod detachment force, N.

According to geometric relation, considering there was backward elastic deformation of the teeth to some extent, there was:

α+β=π (24)

$$\beta +\gamma =\arccos\frac{R+h-H}{R}=1.287\;\mathrm{rad}$$ (25)

Where *R* was the outer diameter of the drum, which was 335 mm; *h* was the minimum drum-ground distance during operation, which was 110 mm; *H* was the minimum drum-ground distance when the teeth started to hull soybeans, which was 123 mm, as shown in Table 2.

Substitute Eqs (21) to (25) into Eq (20), the following equation can be obtained after calculation:

$$\begin{array}{l} \mu g\sin(\beta +\gamma )+\mu R\omega^{2}\sin(\pi -\alpha )+\mu F_{t}\\ \geq g\sin(\beta +\gamma )+R\omega^{2}\cos(\pi -\alpha ) \end{array}$$ (26)

The *μ* value was obtained through the tilting table test on green soybeans [25], and the value is 0.5. It can be obtained that the bending angle *α* of the picking elastic claw was 2.88 rad.

$$R=l_{1}+l_{2}\cos(\pi -\alpha )$$ (27)

*l*_2_ was decided by the length of the pod 4 cm, and *l*_1_ was 30 cm.

**Fig 12:**
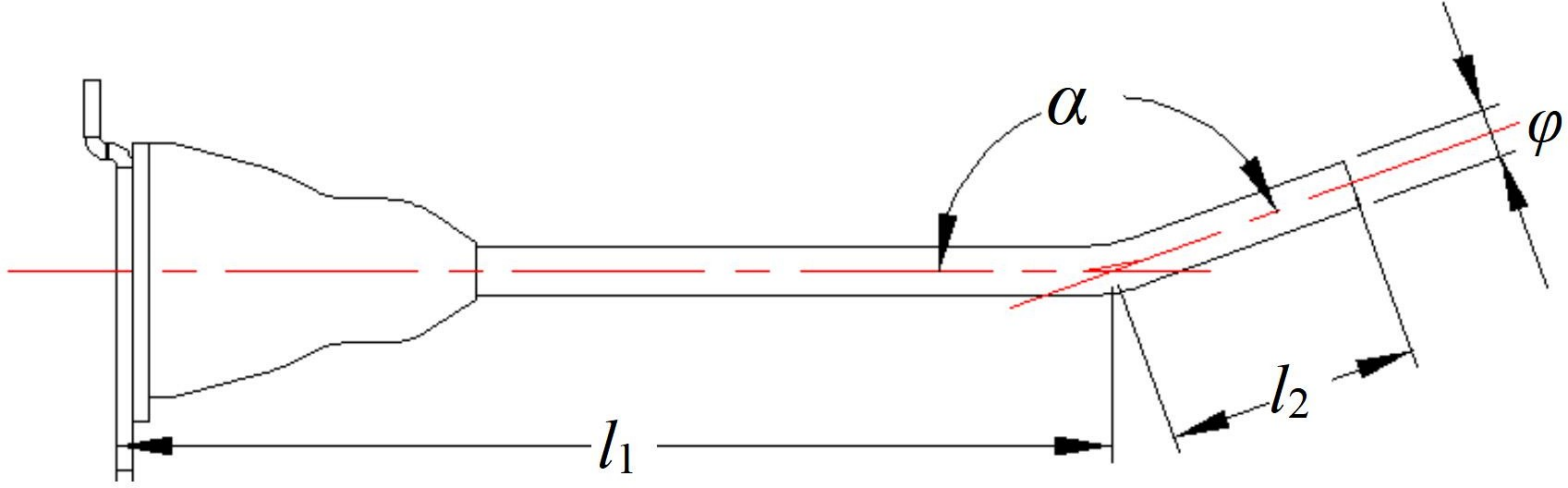
Structure of the elastic tooth. Note: *α* is the bending angle of the tooth, (rad); *l*_1_ is the distance between the bending point of the finger and the base, mm; *l*_2_ is the length of the bending section of the tooth, mm; *φ* is the diameter of the tooth, mm.

The structure of an elastic tooth is shown in Fig 12. The whole elastic tooth was made up of a pop-up finger, a rubber base, and a connecting plate. The connecting plate was molded by casting and was riveted uniformly to the drum; the elastic tooth was in integral casting with the rubber base for damping and avoiding resilience in high-speed collision between teeth and soybean stalks and pods in harvesting, so that the damage rate of pods can be reduced and the teeth would not be ruptured easily. The comb teeth on the drum were designed and arranged according to the principle of uniform and non-missing combing.

**Fig 13:**
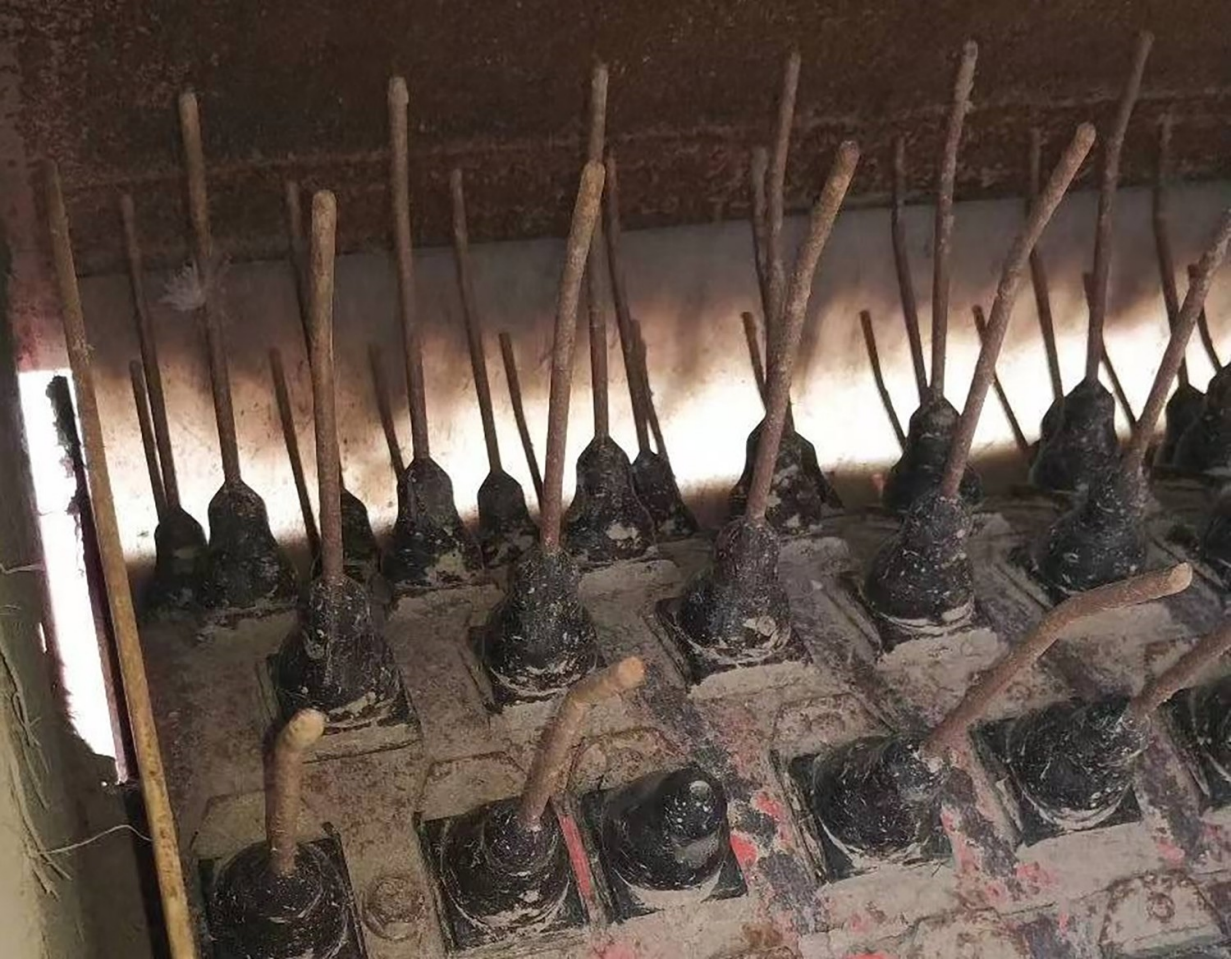
Practicality picture of the drum teeth.

The functions of the comb teeth were separation, conveying, and prevention of pod drop. Each comb tooth had a diameter of 8mm, and the inclination angle at the end was 2.88. If the angle was too large, the pods were prone to falling to the ground, and if it was too small, the pods were likely to be carried back and unable to be conveyed to the conveyor belt. The practical picture of the drum teeth is shown in Fig 13.

### Harvest quality index and determination method

The measuring device was selected referring to NY/T995-2006 Operation Quality of Grain (Wheat) Combine Harvesting Machinery, and the following standards were taken for detection of the working performance of the harvester: pod detachment rate ≥ 90%, pod damage rate ≤ 5% [25].

After completing the experiment, the mass of the pods on the ground, the mass of the pods remaining on the plants, and the mass of the pods collected in the grain box were collected and measured. The pod detachment rate of the drum equipment can be expressed using Eq 28.

$$Y_{1}=\frac{W_{L}}{W_{L}+W_{Z}}\times 100\%$$ (28)

Where, *Y*_1_ was the pod detachment rate, %; *W*_*L*_ was the mass of the pods in the grain box, kg; *W*_*Z*_ was the total mass of pods on the ground and the plants, kg.

To calculate the pod damage rate after harvesting, randomly take out 50 kg pods in the grain box collect the damaged pods, and weigh them. The pod damage rate was expressed in Eq 29.

$$Y_{2}=\frac{W_{U}}{50}\times 100\%$$ (29)

Where, *Y*_*2*_ was the pod damage rate, %; *W*_*U*_ was the mass of damaged pods, kg.

## Analysis of performance test

### Test materials

**Fig 14:**
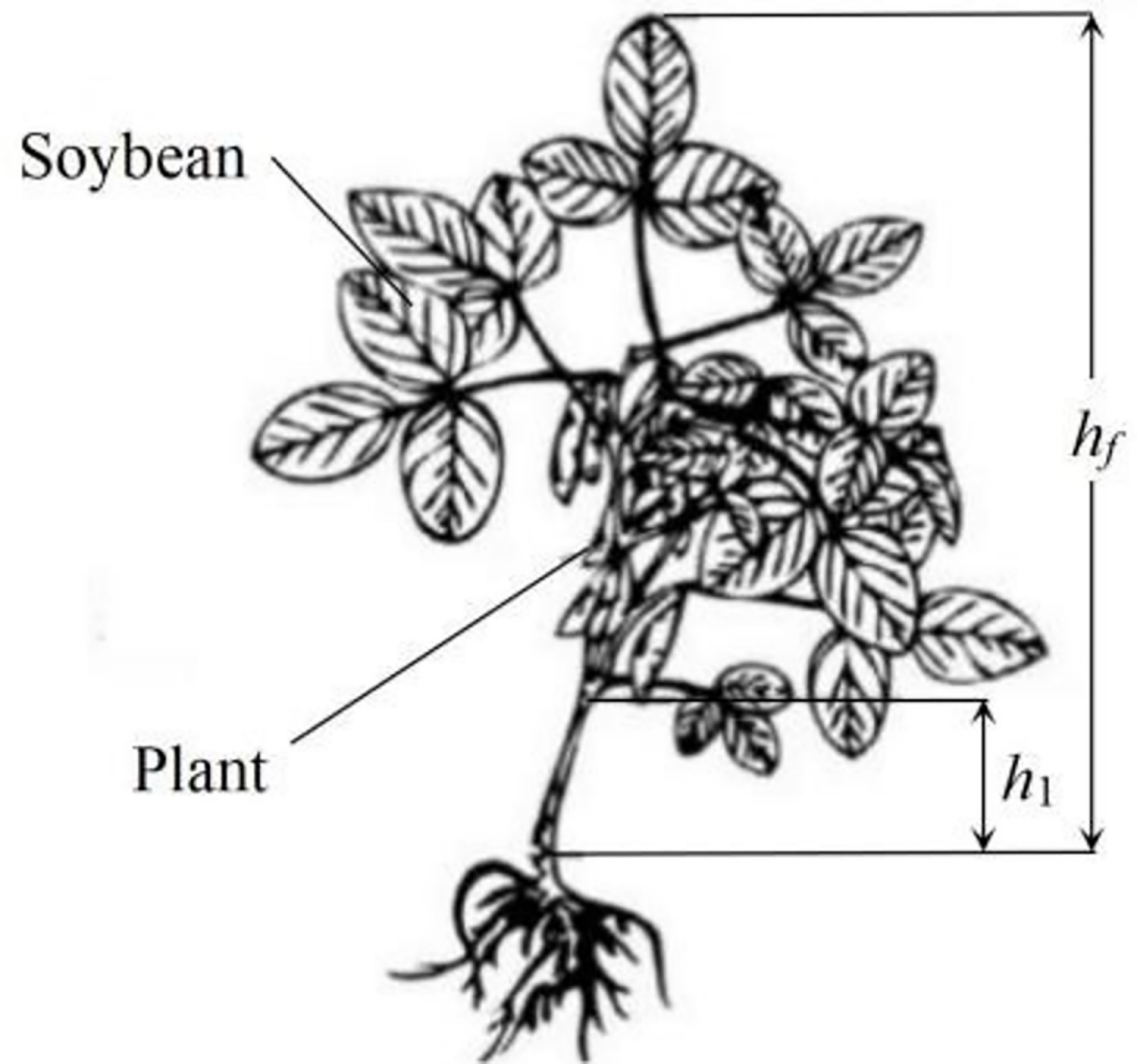
Green soybean plants. Note: *h*_*f*_ was plant height; *h*_2_ was the height of the soybean pod from the ground.

To improve the working performance of the self-propelled green soybean harvester, an optimization test was carried out on its working parameters. The field test was carried out at the green soybean planting base of Jiangsu Yanjiang Institute of Agricultural Sciences on October 25, 2019, in China. Green soybean varieties of ‘Xiao Nong Qiu Yan’ and ‘Dou Tong No.6’ were taken as test objects, whose maturity period was generally the last ten days of October, and the schematic diagram of the plant is shown in Fig 14 and the relevant physical characteristics are shown in Table 1.

**Table 1:** Physical characteristics of fresh soybean plants.

Variety	*h*_*f*_ Plant height	*h*_1_ Soybean height from the ground	Stalk diameter	The average weight of the pod	The average number of podding
Xiao Nong Qiu Yan	41.2~65.5	14.3~22.5	4.8~6.5	50.2	18.5
Dou Tong No. 6	37.2~59.8	14.2~25.5	5.2~6.57	50.8	20.3

### Test instrument

**Fig 15:**
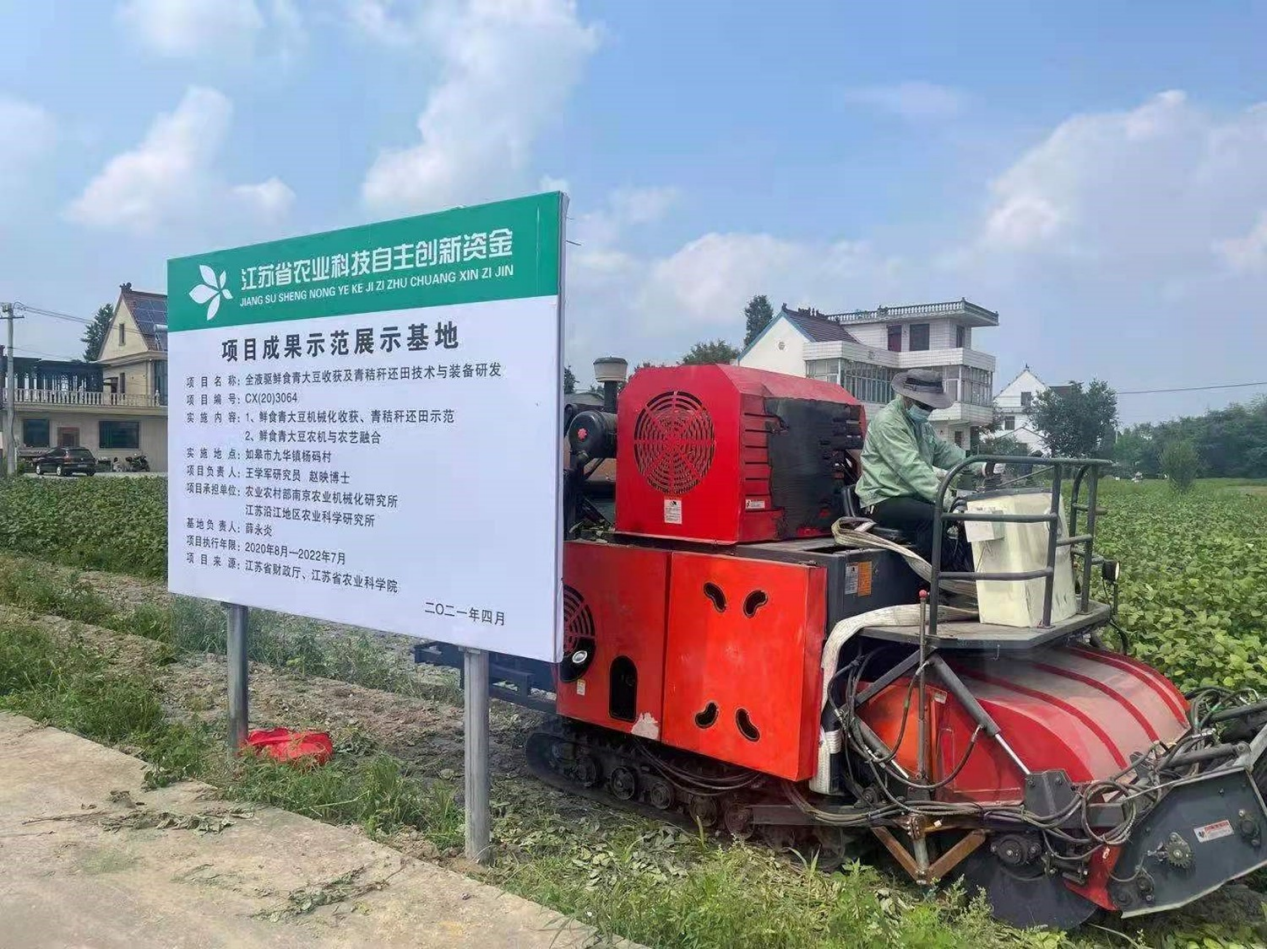
The experimental equipment.

Victor6236P tachometer, with an accuracy of 0.05%+1; measuring tape: measuring range of 100 m, with an accuracy of 0.01 m; SC900 soil hardness tester, with an accuracy of 0.001 kPa; electronic scale: measuring range of 100 kg, accuracy 0.001 kg; MS-10 soil moisture speedometer, with an accuracy of 0.1%. The experimental equipment employed is depicted in Fig 15.

### The single-factor test of the detachment performance

To further study the effects of the parameters including the rotational speed of drum teeth *A*_1_, axial minimum teeth distance *A*_2,_ and travel speed *A*_3_, the single factor test method was used to analyze the influence of each parameter in turn on the detachment rate and damage rate. Each test was replicated three times. According to the common technical parameters of field harvesting, the test parameters selected were set as follows:

The general rotational speed of drum teeth *A*_1_ was 280–500 rpm/min [17]. Too high rotational speed would cause excessive force to damage the soybean pods, and too low rotational speed would lead to too small force of comb-brushing to miss collection. Considering the above reasons, the test parameters of *A*_1_ were set as 280, 340, 380, 440, and 500 rpm/min by adjusting the main hydraulic motor of the harvester.

The general axial minimum teeth distance *A*_2_ was 3.5–4.8 mm. Too large an axial minimum teeth distance can easily cause some pods not to be brushed, resulting in missed picking. Too small an axial minimum teeth distance can easily cause pods to be brushed repeatedly, resulting in higher impurity content and damage rate. Considering the above reasons, the minimum teeth distance *A*_2_ was set as 3.5, 3.8, 4.1, 4.5, and 4.8 mm.

The general travel speed *A*_3_ was 0.35–0.85 m/s, and then the test parameters of *v* were set as 0.35, 0.5, 0.65, 0.7, and 0.85 m/s by adjusting the chassis traveling hydraulic motor.

#### Effect of the rotational speed of drum teeth on harvest quality

**Fig 16:**
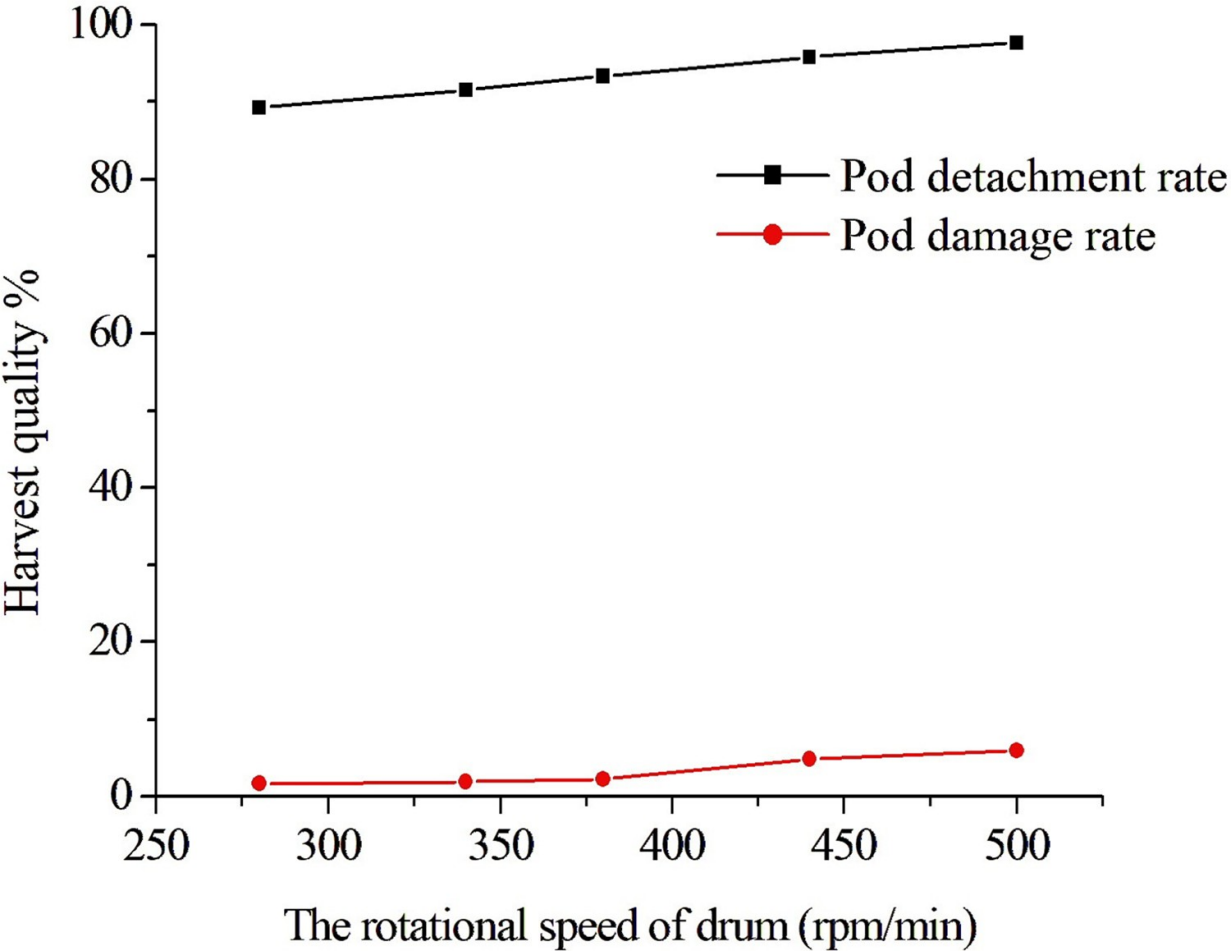
The harvest quality situation under different drum rotational speeds.

The axial minimum teeth distance *A*_2_ was 4.5 mm, the travel speed *A*_3_ was 0.65 m/s, and the rotational speed of drum teeth was 280, 340, 380, 440, and 500 rpm/min in this test. Fig 16 shows the harvest quality situation at different rotational speeds. It can be seen that the faster the rotational speed was, the greater the impact of the brush on the fresh green soybean pods. The pod detachment rate increased with the increase of the rotational speed, but the pod damage rate also increased with the increase of the rotational speed. It can also be seen that with the increase in the rotational speed, the highest pod detachment rate was 97.6%, and the damage rate was also as high as 5.97%. It should be noted that the damage rate only counts the appearance damage, and the internal seed quality cannot be counted, which would cause further waste in transportation. However, under the test of the lowest rotational speed, the highest pod detachment rate was only 89.2%. The above results showed that the rotational speed had a great impact on the harvest quality. To obtain a higher pod detachment rate and lower pod damage rate, it is necessary to perform further study.

#### Effect of the axial minimum teeth distance on harvest quality

**Fig 17:**
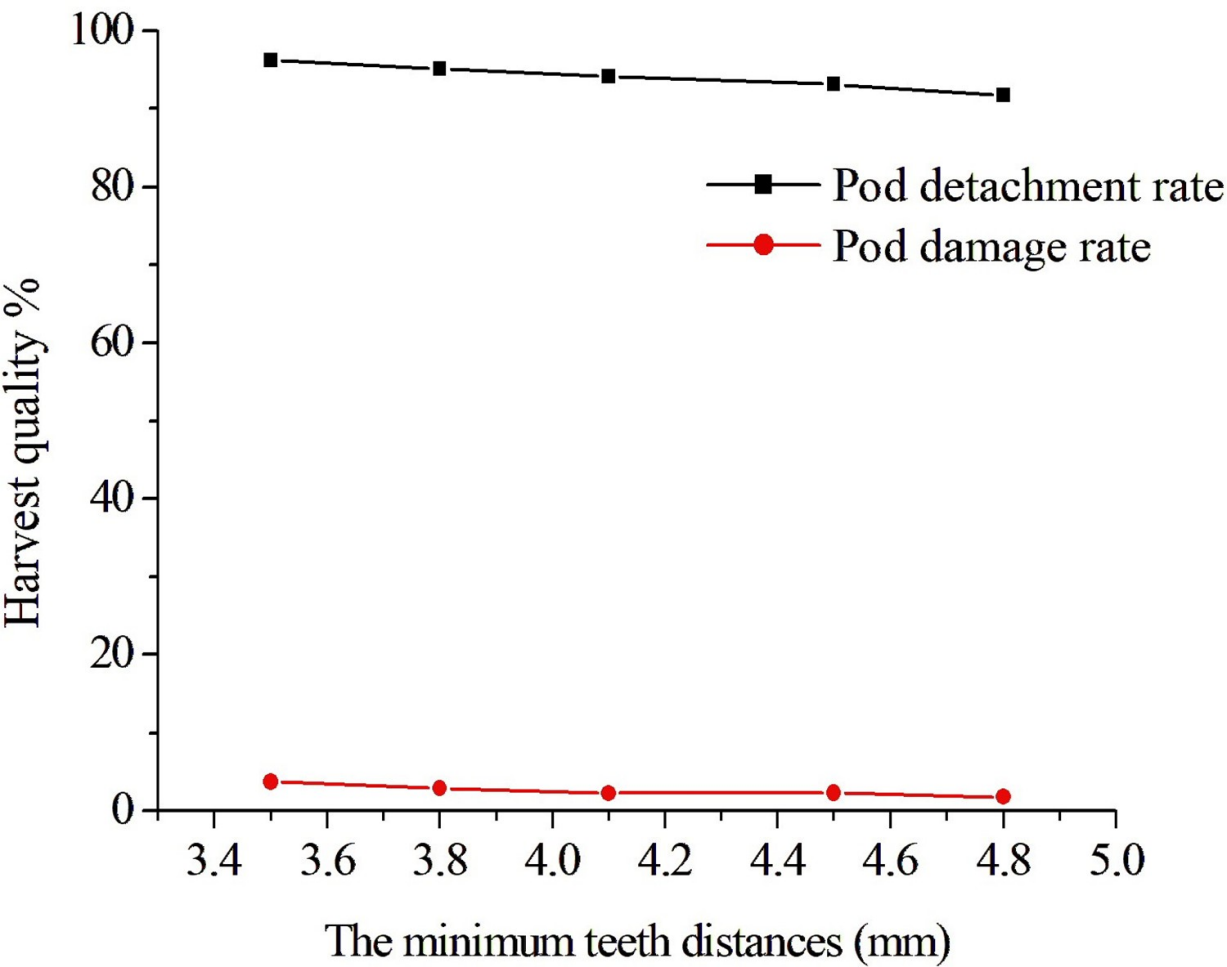
The harvest quality situation under different axial minimum teeth distances.

The rotational speed *A*_1_ was 380 rpm/min, the travel speed *A*_3_ was 0.65 m/s, and the axial minimum teeth distance *A*_2_ were 3.5, 3.8, 4.1, 4.5, and 4.8 mm in this test. Fig 17 shows the harvest quality situation at different axial minimum teeth distances. It can be seen that the larger the axial minimum teeth distance was, the less the number of pod collisions during one revolution of the harvester, and the pod detachment rate and damage rate would decrease with the increase of the axial minimum teeth distance. It can be seen that the axial minimum teeth distance was increased, the pod detachment rate was reduced to 91.7%, and the pod damage rate was also reduced to 1.81%; when the axial minimum teeth distance was reduced, the pod detachment rate increased to 96.2%, and the pods damage rate also increased to 3.78%. The above results showed that the rotational speed had also a great impact on the harvest quality. We found that as long as the axial minimum teeth distance was less than the short axis distance of the soybean pods, the pods may be affected by the force of the comb-brush teeth during one revolution of the harvester, which can be listed as the second factor and needed further optimization.

#### Effect of travel speed on harvest quality

**Fig 18:**
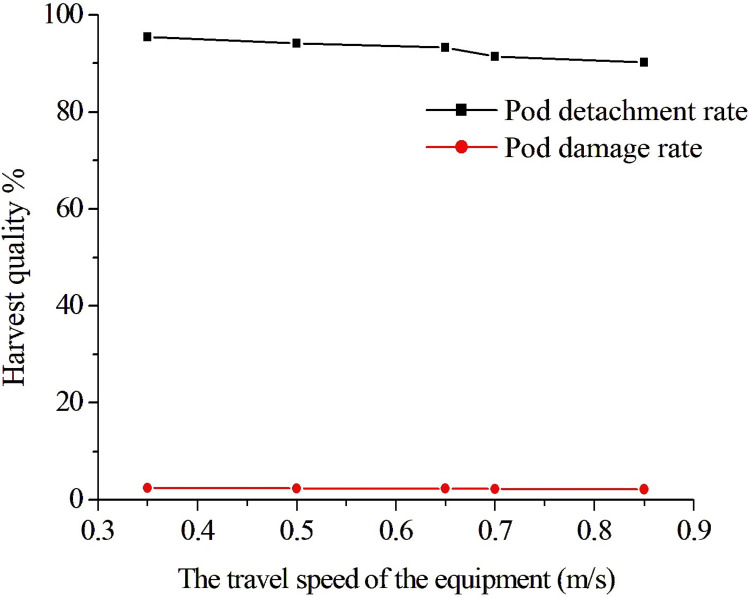
The harvest quality situation under different travel speeds.

The rotational speed *A*_1_ was 380 rpm/min, the axial minimum teeth distance *A*_2_ was 4.5 mm, and the travel speed *A*_3_ was 0.35, 0.5, 0.65, 0.7, and 0.85 m/s in this test. Fig 18 shows the harvest quality situation at different travel speeds. It can be seen that the higher the travel speed was, the less the number of pod collisions during one revolution of the harvester, and the pods detachment rate decreased and the impact of pods damage rate was low with the increase of the travel speed. It can be seen that with the increase of the travel speed, the pod detachment rate and pod damage rate decreased to 90.2% and 1.81% respectively; when the travel speed decreased, the pod detachment rate and pod damage rate increased to 95.4% and 3.51% respectively. The above results showed that the travel speed had a significant effect on detachment quality and needed further optimization.

The rotational speed of drum teeth *A*_1_, the axial minimum teeth distance *A*_2_, and the travel speed *A*_3_ have certain effects on the harvest quality. The above tests provide a good reference value. However, there are constraints among the proposed factors, and it is necessary to achieve the goal of higher travel speed, higher pod detachment rate, and lower loss rate in practice. The single-factor test obviously can not get the best result, and it needs further optimization.

### Multifactor test

#### Experimental method design

The method of quadratic regression orthogonal rotation combination optimization test of three factors and five levels was adopted for regression analysis. In the test, the rotational speed of teeth *A*_1_, the minimum axial teeth distance *A*_2_, and the travel speed of the machine *A*_3_ were selected as the test factors, and the pod detachment rate *Y*_1_ and pod damage rate *Y*_2_ were selected as the optimization objective [22]. A total of 17 groups of tests were carried out, and each group was repeated 3 times, the mean values of the three tests were taken as test results. The test factors and their level codes, test scheme, and results are shown in Tables 2 and 3 respectively.

**Table 2:** Test factors and levels.

Codes	Test factors		
	The rotational speed of the drum *A*_1_ / (rpm/min)	Minimum teeth distance in axial direction *A*_2_ / (mm)	Travel speed of the device *A*_3_ / (m/s)
1.682	500	4.8	0.85
1	440	4.5	0.7
0	380	4.1	0.65
-1	340	3.8	0.5
-1.682	280	3.5	0.35

**Table 3:** Test scheme and results.

Test No.	The rotational speed of the drum *A*_1_ / (rpm/min)	Minimum teeth distance in axial direction *A*_2_ / (mm)	Travel speed of the device *A*_3_ / (m/s)	Pods detachment rate *Y*_1_ / (%)	Pods damage rate *Y*_2_ / (%)
1	500	4.15	0.50	96.7	5.97
2	390	4.15	0.68	94.2	4.23
3	390	3.50	0.85	95.1	4.57
4	280	4.15	0.85	90.4	1.48
5	390	4.80	0.85	93.1	3.21
6	500	4.15	0.85	96.4	5.81
7	390	4.80	0.50	93.8	2.43
8	390	4.15	0.68	92.7	2.31
9	280	3.50	0.68	91.2	1.72
10	390	4.15	0.68	92.4	2.32
11	390	4.15	0.68	92.9	2.34
12	500	3.50	0.68	97.2	6.37
13	280	4.80	0.68	88.8	1.41
14	390	3.50	0.50	94.5	4.23
15	500	4.80	0.68	95.8	5.82
16	280	4.15	0.50	90.4	1.57
17	390	4.15	0.68	93.8	4.19

The variance analysis was conducted by the software Design Expert [26], as shown in Table 4, and the coded regression mathematical models were obtained with pod detachment rate and damage rate as response functions and influencing factors as independent variables, as shown in Eqs (30 and 31).


$$\begin{array}{l} Y_{1}=103.048+0.03621A_{1}-7.5608A_{2}-12.256A_{3}+\\ 3.4965\times 10^{-3}A_{1}A_{2}-3.896\times 10^{-3}A_{1}A_{3}-2.857A_{2}A_{3}-\\ 2.479\times 10^{-5}A_{1}^{2}+0.828A_{2}^{2}+18.776A_{3}^{2} \end{array}$$ (30)

$$\begin{array}{l} Y_{2}=20.819-3.06852\times 10^{-3}A_{1}-7.527A_{2}-12.0629A_{3}\\ -8.39\times 10^{-4}A_{1}A_{2}-9.091\times 10^{-4}A_{1}A_{3}+0.967A_{2}A_{3}+\\ 3.51\times 10^{-5}A_{1}^{2}+0.7746A_{2}^{2}+6.686A_{3}^{2} \end{array}$$ (31)

**Table 4:** Results of variance analysis.

Test indexes	Sources of variance	Sum of squares	Degree of freedom	Mean square	*F*	*P*
*Y*_1_/%	Model	88.27	9	9.81	26.84	0.0001
	Residual error	2.56	7	0.37		
	Lack-of-fit	0.22	3	0.073	0.12	
	Error	2.34	4	0.58		
*Y*_2_/%	Model	43.27	9	4.81	6.49	0.01
	Residual error	5.1	7	0.74		
	Lack-of-fit	0.91	3	0.30	0.28	
	Error	4.27	4	1.07		

Where, *A*_1_ was the rotational speed of the teeth, rpm/min; *A*_2_ was the minimum axial teeth distance, mm; *A*_3_ was the travel speed of the device, m/s; variance analysis was conducted on pod detachment rate *Y*_1_ and damage rate *Y*_2_ and the test results were shown in Table 4. The results of the regression equation model showed that the pod detachment rate *Y*_1_ and damage rate *Y*_2_ were *P*< 0.0001 and *P*< 0.01 respectively, indicating that the *F* test of *Y*_1_ and *Y*_2_ models were significant, and the two regression equation models were significant.

#### Interaction effect of test factors on pod detachment rate

**Fig 19:**
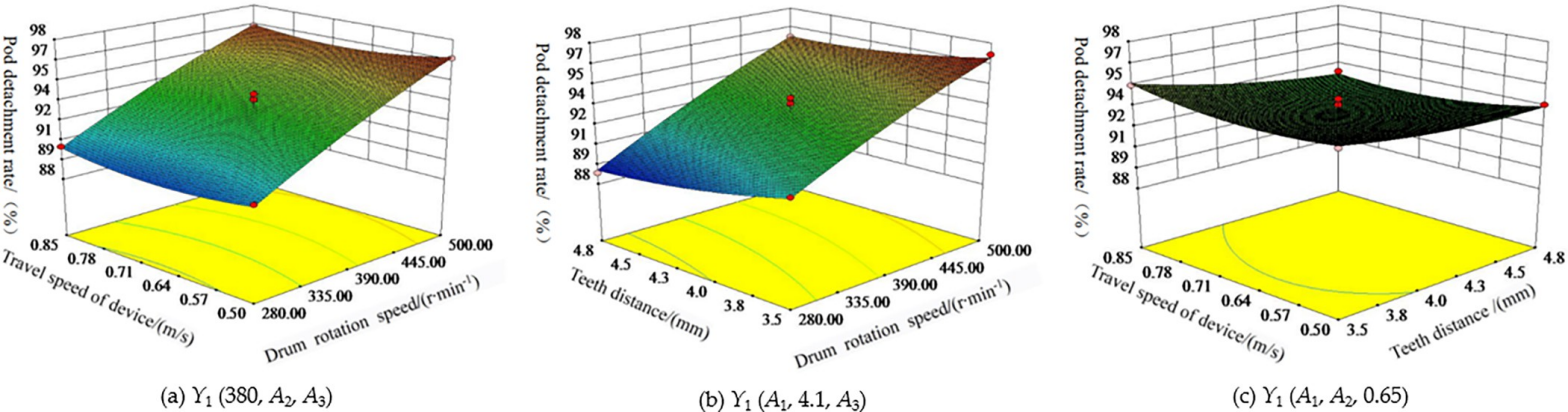
Influence of test factors on pods detachment rate *Y*_1_. (a) *Y*_1_ (380, *A*_2_, *A*_3_). (b) *Y*_1_ (*A*_1_, 4.1, *A*_3_). (c) *Y*_1_ (*A*_1_, *A*_2_, 0.65).

When the rotational speed of the teeth *A*_1_, the minimum axial teeth distance *A*_2_, and the travel speed *A*_3_ of the machine were fixed at zero level (i.e. *A*_1_ = 380 rpm/min, *A*_2_ = 4.1 mm, *A*_3_ = 0.65 m/s), the interaction effect of *A*_2_ and *A*_3_, *A*_1_ and *A*_3_, *A*_1_ and *A*_2_ on the pod detachment rate was shown in Fig 19(A)–19(C) respectively. The pod detachment rate *Y*_1_ increased in the positive response to the rotational speed of the teeth *A*_1_, while the teeth distance *A*_2_ and the travel speed of the device *A*_3_ increased in a negative response. Among them, the rotational speed of the teeth *A*_1_ changed rapidly on the response surface, the teeth distance *A*_2_ changed moderately on the response surface, and the travel speed *A*_3_ changed slowly on the response surface. The rotational speed of teeth *A*_1_ had the most significant effect on the pod detachment rate *Y*_1_, followed by the minimum axial teeth distance *A*_2_ and the travel speed of the device *A*_3_.

### Analysis of the influence of interaction effect of test factors on pod damage rate

**Fig 20:**
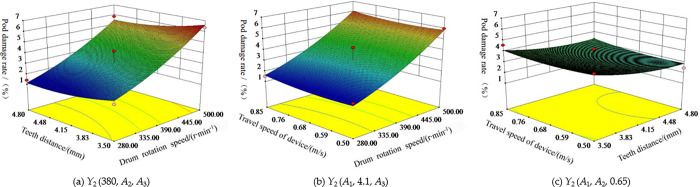
Influence of interaction effect of test factors on damage rate *Y*_2_. (a) *Y*_2_ (380, *A*_2_, *A*_3_). (b) *Y*_2_ (*A*_1_, 4.1, *A*_3_). (c) *Y*_2_ (*A*_1_, *A*_2_, 0.65).

When the rotational speed of the teeth *A*_1_, the minimum axial teeth distance *A*_2_, and the travel speed *A*_3_ of the machine were fixed at zero level (i.e. *A*_1_ = 380 rpm/min, *A*_2_ = 4.1 mm, *A*_3_ = 0.65 m/s), the interaction effect of *A*_2_ and *A*_3_, *A*_1_ and *A*_3_, *A*_1_ and *A*_2_ on the damage rate is shown in Fig 20(A)–20(C) respectively. The damage rate *Y*_2_ increased in the positive response to the rotational speed of the teeth *A*_1_, while the teeth distance *A*_2_ and the travel speed of the device *A*_3_ increased in a negative response. Among them, the rotational speed of the teeth *A*_1_ changed rapidly on the response surface, the teeth distance *A*_2_ changed moderately on the response surface, and the travel speed *A*_3_ changed slowly on the response surface. The rotational speed of teeth *A*_1_ had the most significant effect on the pod damage rate *Y*_2_, followed by the minimum axial teeth distance *A*_2_ and the travel speed of the device *A*_3_.

#### Comprehensive influence analysis

Through comprehensive analysis, it can be found that higher pod detachment rate and higher pod damage rate co-exist at the same time. The overall impact tendency of the rotational speed of teeth, the minimum axial teeth distance, and the travel speed of the machine on pod detachment rate and damage rate is the higher the rotational speed of the teeth, the smaller the distance of them, the lower the travel speed of the device, the higher the pod detachment rate, and the higher the pod damage rate. The reason for this phenomenon is that, during the interaction of teeth and green soybean plants, the collision from the high-speed rotational of teeth on the pods would brush and hull the pods, and at the same time, damage the pods. The higher the rotational speed of the teeth, the higher the linear velocity of the collision between the teeth and green soybeans, thus the higher the impact force to hull the pods more easily, however, in this case, the more easily the pods get damaged. The lower the teeth distance and the travel speed of the device, the more the collisions between the teeth and green soybean pods, thus, the higher the pod detachment rate and damage rate were obtained. Based on the above analysis, it can be inferred that an increase in drum speed, a reduction in comb tooth spacing, and a decrease in equipment travel speed would result in a proportional increase in pods impact force and frequency, leading to a significant rise in pod damage rate. However, in practical field operations, our objective was to achieve a higher pod detachment rate while maintaining a lower pod damage rate.

#### Parameter analysis

In this paper, by taking the high pod detachment rate and low damage rate as the optimization objective, the key parameters of the self-propelled green soybean harvester were optimized. The software Design-Expert was used to establish the quadratic regression optimization analysis model of the two indexes. The constraint condition includes: 1) objective function: *Y*_1_→*Y*_1max_≥94%; *Y*_2_→*Y*_2min_≤4%; 2) Constraint condition of influencing factors: *A*_1_∈[−1,1], the rotational speed of the teeth is 340~440 r·min^-1^; *A*_2_∈[−1,1], the minimum axial teeth distance is 3.8~4.5 mm; *A*_3_∈[−1,1], the travel speed of the device is 0.5~0.7m/s. The optimal parameters are as follows: when the rotational speed of the drum was 397.36 rpm/·min, the minimum axial teeth distance was 4.8 mm, and the travel speed of the equipment was 0.5 m/s, the predicted pod detachment rate of the model was 94%, and the pod damage rate was 3.04%.

### Field verification test on the optimized parameters

**Fig 21:**
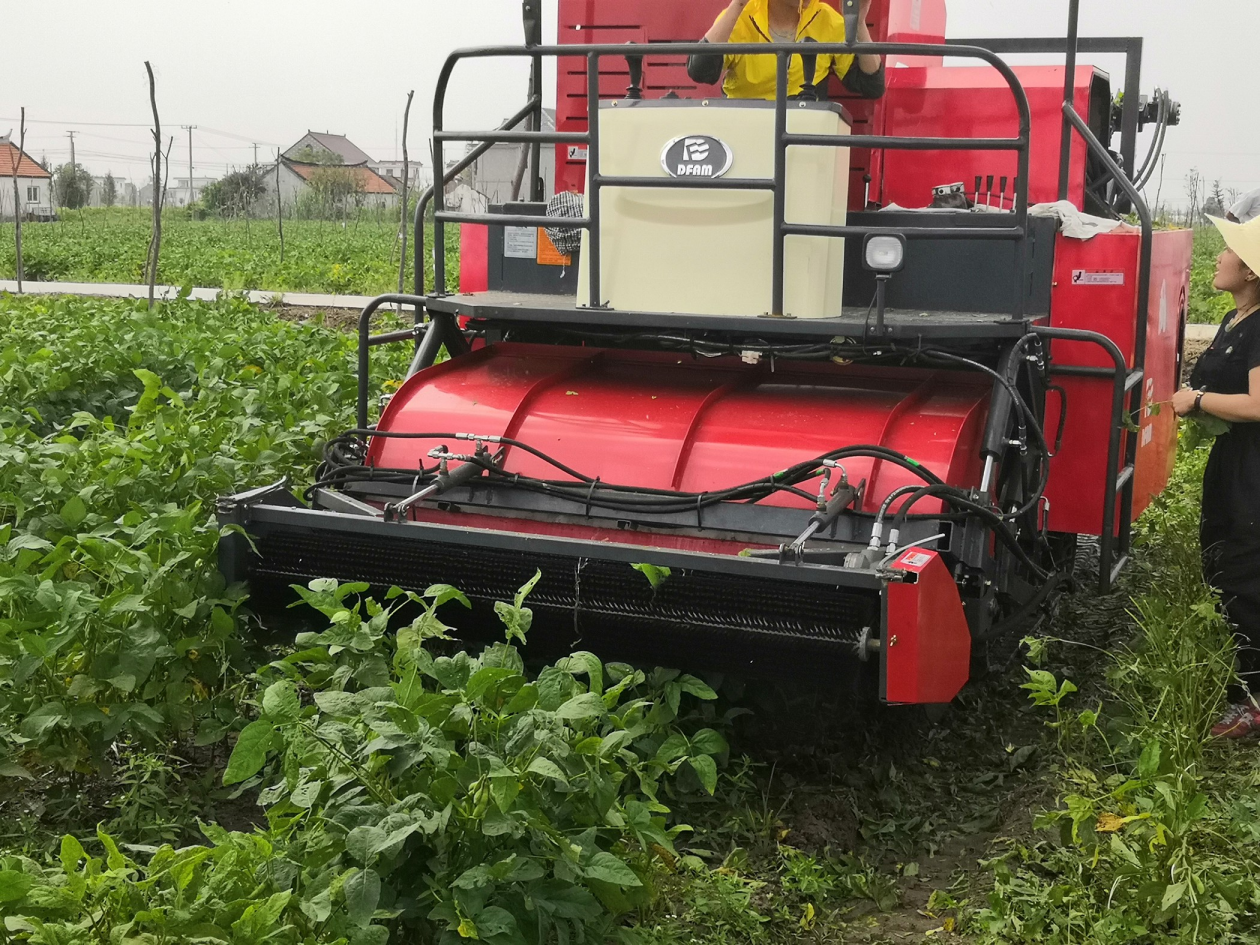
Field test of 4GQD-160 type self-propelled green soybean harvester.

**Fig 22:**
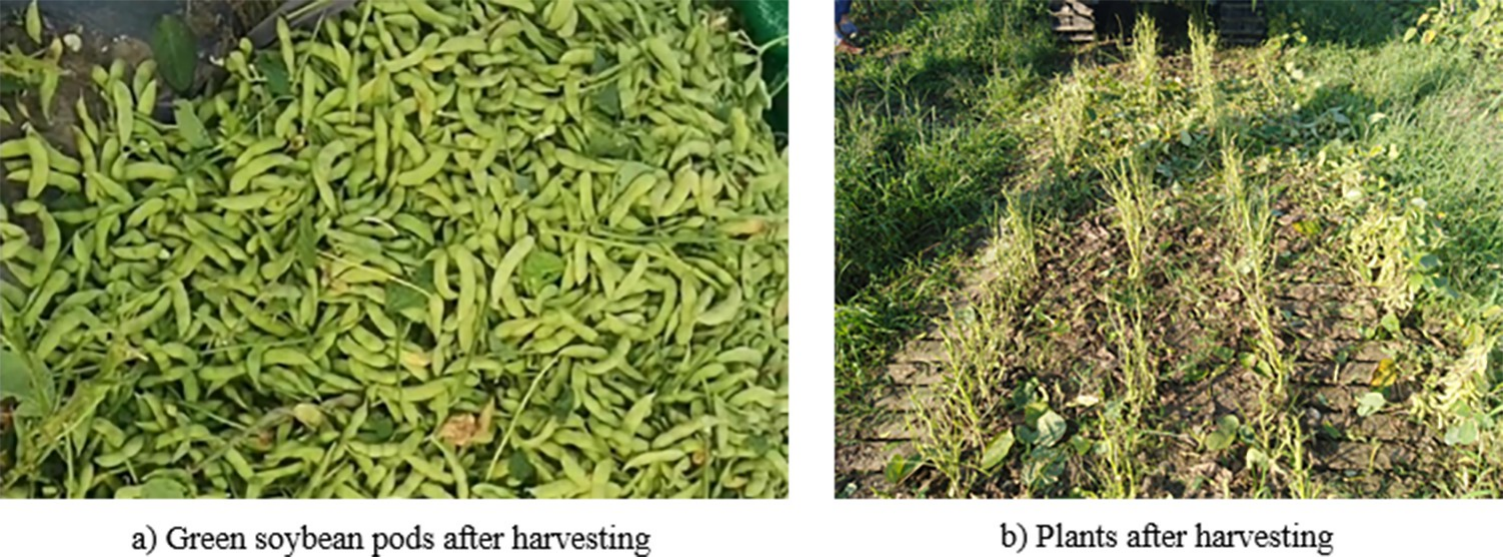
Green soybean pods and plants after harvesting. a) Green soybean pods after harvesting. b) Plants after harvesting.

The verification test was conducted on June 28, 2020, in Hengtang Vegetable Base of Jiangsu Changshu Bixi Export Vegetable Demonstration Park and Green Soybean Planting Base of Jiangsu Yanjiang Institute of Agricultural Sciences, as shown in Figs 21 and 22.

The following parameter combination in the field verification test was adopted: the rotational speed of the teeth was 397.36 rpm/min, the minimum axial teeth distance was 4.8 mm, and the travel speed of the device was 0.5 m/s. The test was carried out in 5 groups, and mean values were taken as test results, as shown in Table 5. Results of field verification test on harvesting of ‘Xiao Nong Qiu Yan’ and ‘Dou Tong No. 6’ by the 4GQD-160 type self-propelled green soybean harvester show that, under the parameter combination above, the harvesting efficiency˃0.187 hm^2^/h, detachment rate˃91%, damage rate<4.5% and content impurity rate<7.8%, The test results show that the operation effect of the combined machine met the design requirements.

**Table 5:** Statistics of the operation process of the harvester.

Variety	Items	Measuring points					Mean value
		1	2	3	4	5	
Xiao Nong Qiu Yan	Lowest podding height from the ground/cm	9.8	10	7.8	5.9	11.5	9.0
	Harvesting efficiency/hm^2^.h^-1^	0.186	0.187	0.186	0.187	0.188	0.187
	Pod detachment rate/%	91.4	91.7	92.1	91.5	91.3	91.6
	Damage rate/%	4.2	4.4	4.6	4.5	4.7	4.48
	Impurity content/%	9.8	7.5	6.7	7.8	6.4	7.64
Dou Tong No.6	Lowest podding height from the ground/cm	13.7	14.2	10.4	12.3	10.5	12.22
	Harvesting efficiency/hm^2^.h^-1^	0.187	0.189	0.187	0.188	0.189	0.188
	Pod detachment rate/%	90.8	91.2	91.3	90.5	90.7	90.9
	Damage rate/%	3.8	3.9	4.1	4.2	3.7	3.94
	Impurity content/%	8.7	7.5	6.9	7.4	8.4	7.78

## Conclusions

(1) Given the problem in mechanized harvesting of green soybeans, in this paper, a kind of self-propelled green soybean harvester was designed, and the key components of the device, a fully hydraulic drive control system, the brushing mechanism on the drum, the hydrostatic walking chassis and the material cleaning device were also designed. The device could complete pod detachment, material delivery, pod cleaning, collection, and unloading at one time and improve the efficiency of mechanized green soybean harvesting to a great extent.

(2) The motion of the pod detachment process was analyzed, and the teeth arrangement principle was proposed, then a front-mounted detachment drum was designed. Through response surface test study, the rotational speed of the teeth, the influence tendency of minimum axial teeth distance, and the travel speed of the device on pod detachment rate and damage rate was analyzed, and a quadratic multinomial response model was established, the results showed that, under each test factor, the influence of rotational speed of the drum on pod detachment rate and damage rate was more significant than the travel speed of the device and the teeth distance.

(3) The software Design-Expert was adopted to optimize the test results, and the optimal parameter combination was obtained as follows: the rotational speed of the teeth was 397.36 rpm/min, the minimum axial teeth distance was 4.8 mm, the travel speed of the device was 0.5 m/s. The predicted pod detachment rate of the model was 94%, and the pod damage rate was 3.04%.

(4) According to the optimal parameter combination, field verification test was conducted, and verification test results on harvesting of ‘Xiao Nong Qiu Yan’ and ‘Dou Tong No. 6’ by the 4GQD-160 type self-propelled green soybean harvester showed that, under the parameter combination above, the harvesting efficiency ˃0.187 hm^2^/h, detachment rate˃91%, damage rate<4.5% and content impurity rate<7.8%. The test results showed that the operation effect of the 4GQD-160 type self-propelled green soybean harvester could meet the design requirements.

### Statement of field test permissions

The green soybean planting base of Jiangsu Yanjiang Institute of Agricultural Sciences provided the field test permission for this study. The green soybeans collected during the test were in the collection room of the test base. At the same time, we confirm that our experimental research and field studies on the green soybean plants, including the collection of plant material, comply with relevant institutional, and international guidelines and legislation in China.

## List of abbreviations

m: The number of drum teeth
n: The number of teeth in the radial direction
a: Distance of two adjacent rows of teeth (mm)
b: The width of a green soybean pod (mm)
*c* (*A*_2_): Minimum teeth distance in the axial direction (mm)
d: The inner diameter of the drum (mm)
r: The radius of the drum (mounting comb teeth) (mm)
R: The outer radius of the drum (mounted comb teeth) (mm)
D: Outer diameter of the drum (2*R*) (mm)
L: Length of drum (mm)
l_s_: Length of a single tooth (mm)
*l*_1_: Distance between the bending section of the teeth and the center of the drum (mm)
l_2_: Length of the bending section of the teeth (mm)
φ: Diameter of the tooth (mm)
h_f_: Height of soybean stalk (m)
*h*_1_: Lowest distance from the ground to soybean (cm)
h: The distance of the drum to the ground (cm)
H: Drum-ground distance when the teeth start to hull soybeans (mm)
H_g_: Distance of drum axis from the ground (cm)
δ: Radial distance of two neighboring teeth after unfolding the drum (mm)
*ω* (*A*_1_): Rotational speed of drum teeth (rpm/min)
*n*_z_: Rotating speed of the drum (rpm/min^-1^)
v_g_: Linear velocity of the teeth on the drum (m/s)
θ: Rotation angle of the drum (rad)
*v* (*A*_3_): Travel speed of the device (m/s
F_f_: Friction when throwing back the green soybeans (N)
F_r_: The centrifugal force of the soybean pods (N)
F_n_: Collision force from teeth to pods (N
F_P_: Pod damage force (N)
G: Gravity of pods (N)
β: The angle between the bending section of the comb and the centrifugal force (rad)
γ: The angle between the gravity of pods *G* and the centrifugal direction of *F*_*r*_ (rad)
α: Bending angle of the tooth (rad)
*α*_1_: The angle between ground and plant before harvest (rad)
*α*_2_: The angle between plants and ground in the feeding area (rad)
*α*_3_~*α*_4_: The angle between plants and ground in the harvest area (rad
*α*_5_: The angle between plants and ground in the extraction state (rad)
vt: *vt* is the advance distance of *C* to *E* (m)
Δ*t*: Contact time between teeth and pods(s)
W_L_: Total weight of pods in the grain tank (kg)
W_Z_: Total weight of pods on the ground and the plants (kg)
W_U_: Weight of damaged pods (kg)
*Y*_1_: Pods detachment rate (%)
Y_2_: Pods damage rate (%)
*a*_1_, *a*_2_, *a*_3_,…, *a*_*n*_: The first column in the radial direction of the drum
*b*_1_, *b*_2_, *b*_3_,…, *b*_*n*_: The second column in the radial direction of the drum
*m*_1_, *m*_2_, *m*_3_,…, *m*_*n*_: The m-th columns in the radial direction of the drum
R_m_: The tangential point between the plant and the inner diameter of the roller
R_n_: The tangential point between plant D and the inner circle of the drum
cos*ξ*: The collision loss coefficient of pods during harvesting process
